# Multimodal diagnostics for keratoconus and ectatic corneal diseases: a paradigm shift

**DOI:** 10.1186/s40662-023-00363-0

**Published:** 2023-11-03

**Authors:** Renato Ambrósio, Marcella Q. Salomão, Lorena Barros, João Batista R. da Fonseca Filho, Jaime Guedes, Alexandre Neto, Aydano P. Machado, Bernardo T. Lopes, Nelson Sena, Louise Pellegrino Gomes Esporcatte

**Affiliations:** 1 Department of Ophthalmology, Federal University the State of Rio de Janeiro (UNIRIO), Rio de Janeiro, Brazil; 2 Department of Ophthalmology, Federal University of São Paulo, São Paulo, Brazil; 3 Rio de Janeiro Corneal Tomography and Biomechanics Study Group, Rio de Janeiro, Brazil; 4 Rio Vision Hospital, Rua Prudente de Moraes, 1276, Rio de Janeiro, RJ 22420-042 Brazil; 5 Brazilian Study Group of Artificial Intelligence and Corneal Analysis – BrAIN, Rio de Janeiro & Maceió, Brazil; 6 Benjamin Constant Institute, Rio de Janeiro, Brazil; 7 Computing Institute, Federal University of Alagoas, Maceió, Brazil; 8 Department of Ophthalmology, Alder Hey Children’s Hospital, Liverpool, L12 2AP UK

**Keywords:** Keratoconus, Corneal ectasia, Susceptibility, Multimodal corneal imaging, Corneal biomechanics, Corneal tomography, Refractive surgery

## Abstract

Different diagnostic approaches for ectatic corneal diseases (ECD) include screening, diagnosis confirmation, classification of the ECD type, severity staging, prognostic evaluation, and clinical follow-up. The comprehensive assessment must start with a directed clinical history. However, multimodal imaging tools, including Placido-disk topography, Scheimpflug three-dimensional (3D) tomography, corneal biomechanical evaluations, and layered (or segmental) tomography with epithelial thickness by optical coherence tomography (OCT), or digital very high-frequency ultrasound (dVHF-US) serve as fundamental complementary exams for measuring different characteristics of the cornea. Also, ocular wavefront analysis, axial length measurements, corneal specular or confocal microscopy, and genetic or molecular biology tests are relevant for clinical decisions. Artificial intelligence enhances interpretation and enables combining such a plethora of data, boosting accuracy and facilitating clinical decisions. The applications of diagnostic information for individualized treatments became relevant concerning the therapeutic refractive procedures that emerged as alternatives to keratoplasty. The first paradigm shift concerns the surgical management of patients with ECD with different techniques, such as crosslinking and intrastromal corneal ring segments. A second paradigm shift involved the quest for identifying patients at higher risk of progressive iatrogenic ectasia after elective refractive corrections on the cornea. Beyond augmenting the sensitivity to detect very mild (subclinical or fruste) forms of ECD, ectasia risk assessment evolved to characterize the inherent susceptibility for ectasia development and progression. Furthermore, ectasia risk is also related to environmental factors, including eye rubbing and the relational impact of the surgical procedure on the cornea.

## Background

The development of refractive surgery has led to changing paradigms in different areas of ophthalmology, including the diagnosis and treatment of keratoconus (KC) and ectatic corneal diseases (ECD) [1–3]. In science and philosophy, a paradigm is a conspicuous set of concepts or thought patterns, including theories, research methods, and postulates. A paradigm shift occurs when novel knowledge changes such standards. When evaluating patients with ECD, the concept evolution involves various diagnostic approaches besides the diagnostic confirmation, including screening, categorizing the type of ectasia, staging severity, prognostic assessment, and clinical follow-up (Table 1) [4].

**Table 1:** Diagnostic strategies and imaging tests in ectatic corneal diseases (ECD)

Diagnostic strategies	The Why’s	The How’s
Assess complaints	Recognize the patient’s needs and personal and family history	Anamnesis, visual acuity, slit-lamp exam, and comprehensive ophthalmological exam
Screening	Detect mild forms of ECD and characterize ectasia susceptibility before refractive surgery, contemplating the impact of LVC on the cornea	Placido-disk corneal topography, Scheimpflug tomography, Layered tomography with OCT (or VHF US), and biomechanical assessments
Diagnostic confirmation	Paradigm shift related to the management of ECD and access ectasia risk and progression	Comprehensive clinical evaluation with multimodal imaging
Classification of the type of ECD	Characterize the thinning pattern and pathophysiology of corneal ectasia	Integrating tomographic and biomechanical data with AI, possible future role for genetics and molecular biology
Staging	To assess the severity of the disease before visual loss	Belin´s “ABCD” (tomography) + biomechanical “E” (Homburg) for ectasia/KC staging
Prognostic	Patient counseling with education and management of ECD concerning the disease severity	Tomographic and biomechanical parameters (i.e., SPA-1), patient age, ocular allergy, and eye rubbing habit characterize

*ECD =* ectatic corneal diseases; *LVC =* laser vision correction on the cornea; *OCT =* optical coherence tomography; *VHF-US =* very high-frequency ultrasound; *AI =* artificial intelligence; *KC =* keratoconus; *SPA-1 =* stiffness parameter at the first applanation

Considering the current management possibilities with refractive therapeutic procedures such as crosslinking and intrastromal corneal ring segments (ICRS) implantation [5], the clinician must characterize the disease and its impact on the patient in detail to establish a personalized or individualized treatment strategy. Currently, the treatment of KC and ECD has two objectives: to halt the progression of the disease and vision rehabilitation. Besides the first paradigm shift related to the management of KC and ECD, refractive surgery also determined the need for a more accurate diagnosis of mild forms of KC [6–8]. This is because these cases present a very high risk for iatrogenic ectasia progression after laser vision correction (LVC) procedures [9, 10]. Since the first description of iatrogenic ectasia after laser in situ keratomileusis (LASIK) in a patient considered as forme fruste keratoconus (FFKC) based on the inferior steepening on corneal front curvature maps by Seiler and coworkers [11], the quest for identifying mild forms of KC among refractive candidates is unquestionable [7, 12]. The second paradigm shift is that ahead of increasing sensitivity to detect mild forms of ECD, refractive surgeons must characterize ectasia susceptibility to identify patients at higher risk of progressive iatrogenic ectasia, which also fundamentally concerns the impact of the surgical procedure on the cornea [4, 7, 13, 14].

Placido disk-based corneal topography is sensitive to detecting abnormal front curvature patterns of ectatic disease in patients with relatively normal distance-corrected visual acuity (DCVA) and unremarkable biomicroscopy [7, 12, 15]. However, it is fundamental to consider the need to enhance accuracy for detecting mild or subclinical ectatic disease, given that a relatively normal curvature topography does not exclude mild or early ECD [7, 16]. Multimodal refractive imaging includes diverse technologies besides Placido-disk corneal topography, such as three-dimensional (3D) Scheimpflug tomography, layered or segmental tomography with Bowman's and epithelial thickness mapping by optical coherence tomography (OCT), digital very high-frequency ultrasound (dVHF-US), and ocular wavefront. Furthermore, knowledge and understanding of corneal biomechanics are substantial contributions and significance for enhancing the accuracy of recognizing mild forms of ECD and detecting ectasia progression [6, 17]. Such a plethora of data should be used collaboratively for making conscious decisions, which may be challenging for the clinician.

Artificial intelligence (AI) has proven relevant in integrating the overabundance of data generated to facilitate clinical decisions [6, 17, 18]. We proposed summarizing this concept on the algorithm (A^2^ I)^2^, meaning applied artificial intelligence and applied ancient intelligence. This algorithm pertains to both the philosophical underpinnings of ancient intelligence (the “why”) and the practical application of AI (the “how”). Besides the tremendous evolution of imaging technologies, genetic and molecular biology tests are promising to further increase diagnostic accuracy by allowing personalized treatments [19].

## Diagnostic approaches for ECD

### Screening for ectasia in the general population

In the future, we may eradicate blindness resulting from ectatic diseases or even reduce the burden of such conditions on a population if we establish screening strategies that effectively detect disease in a subclinical or mild phase, allowing for earlier, typically less invasive treatments. For example, a smartphone-based corneal topography system can reach large populations of children and adolescents to screen for KC using AI to classify the data and select patients needing further, more detailed examinations [20].

Methods for identifying patients with mild ectasia disease are relevant because the range of success of less invasive procedures is much lower in patients that present for the first time with advanced disease. In a study in Saudi Arabia, Torres and collaborators identified 4.79% of cases with KC [21]. The screening tests should be noninvasive and demonstrate cost–benefit to identify patients needing more detailed analyses and further treatment, which is a significant concern for the Violet June Global Keratoconus Awareness Campaign [22].

### Classification of ectatic disease

According to Global Consensus from 2015, KC is the most common ECD, and by definition, asymmetric bilateral disease. Moreover, secondary mechanical-related ectasia may occur in only one eye [23] ECD includes a group of disorders characterized by progressive thinning and subsequent protruding of the corneal arrangement [24], including keratoglobus, pellucid marginal degeneration (PMD), and KC. The “thinning location and thinning pattern” are the aspects that distinguish them [23]. Keratoglobus characteristically occurs bilaterally and is categorized by a global widespread thinning and round protrusion of the entire cornea, producing an irregular corneal topography with augmented corneal fragility due to extreme thinning [24]. The thinning is commonly maximal at the periphery and may be up to one-fifth of the average corneal thickness. This condition may be associated with scleral thinning, generating a blue sclera. These findings are visible on slit-lamp examination, particularly in the advanced stages of the disease. However, in uncertain cases, pachymetric maps may allow the clinician to determine the specific region of the cornea presenting the thinning. It is classified as a congenital disorder and is frequently associated with connective tissue diseases; however, current reports propose that keratoglobus may also be developed and related to vernal keratoconjunctivitis atopy, blepharitis, corneal traumas, thyroid eye disease, and extreme eye rubbing [25].

PMD is characterized by a distinctive thin band of corneal thinning near the limbus but conserving a 1–2 mm zone [24]. It is undefined whether these are unique phenotypic variations of KC or, in fact, different disorders. It typically starts later in life and progresses slower than KC. Therefore, corneal topographic indices and the classical crab-claw topographic pattern cannot be used as the primary tool to distinguish between PMD and KC. Scheimpflug imaging-based devices have shown the significance and effectiveness of the pachymetric map for an appropriate diagnosis of PMD. Furthermore, OCT and biomechanical properties have been studied as complementary techniques that may help with diagnosis [26].

**Fig. 1:**
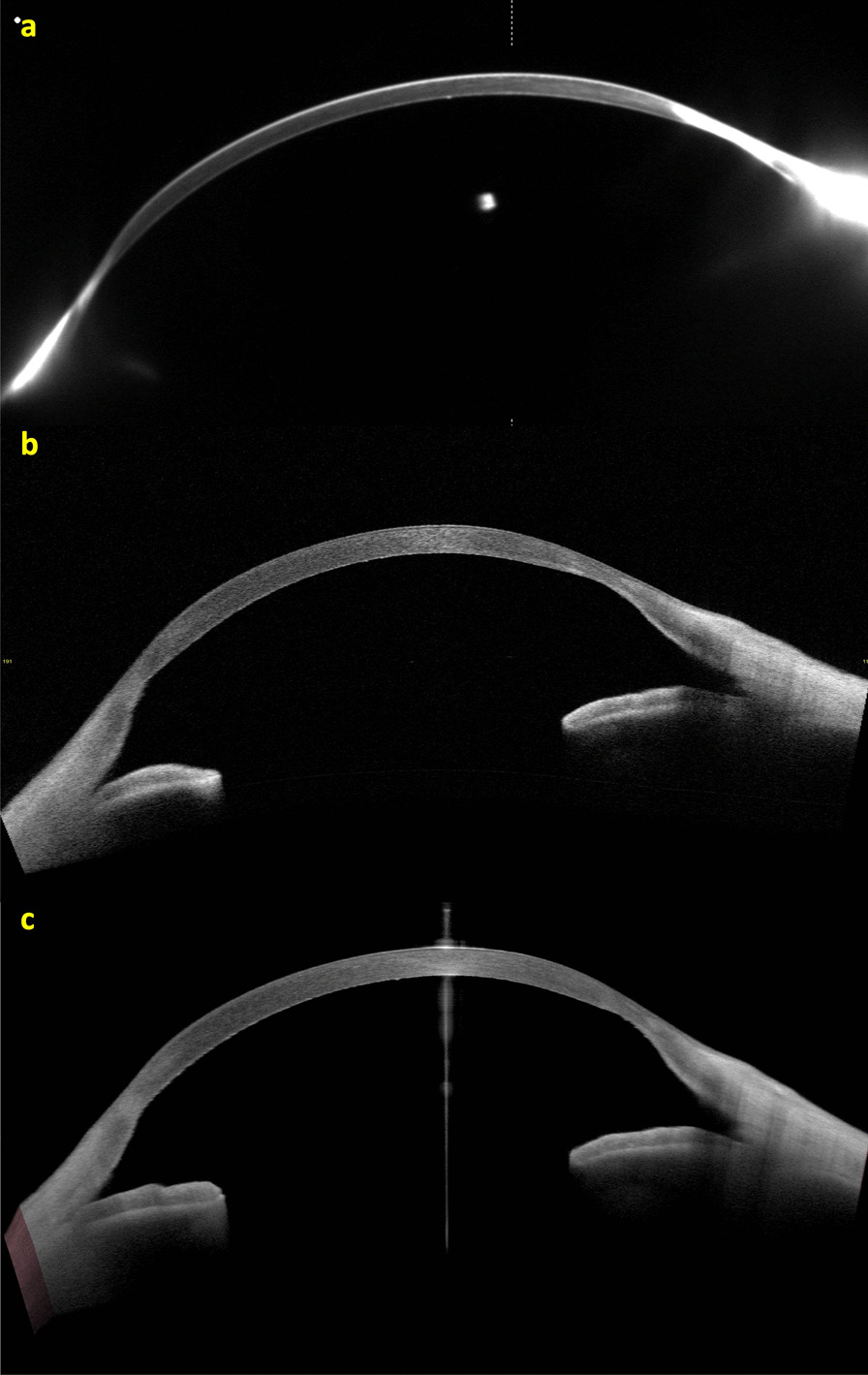
The Scheimpflug image (Pentacam AXL) (**a**), the Tomey Cassia 2 (**b**) and Anterion OCT (Heidelberg Engineering GmbH) (**c**) of the cornea and anterior chamber of the right eye of a 45-year-old patient with traumatic ectasia with a pellucid marginal degeneration thinning pattern

**Fig. 2:**
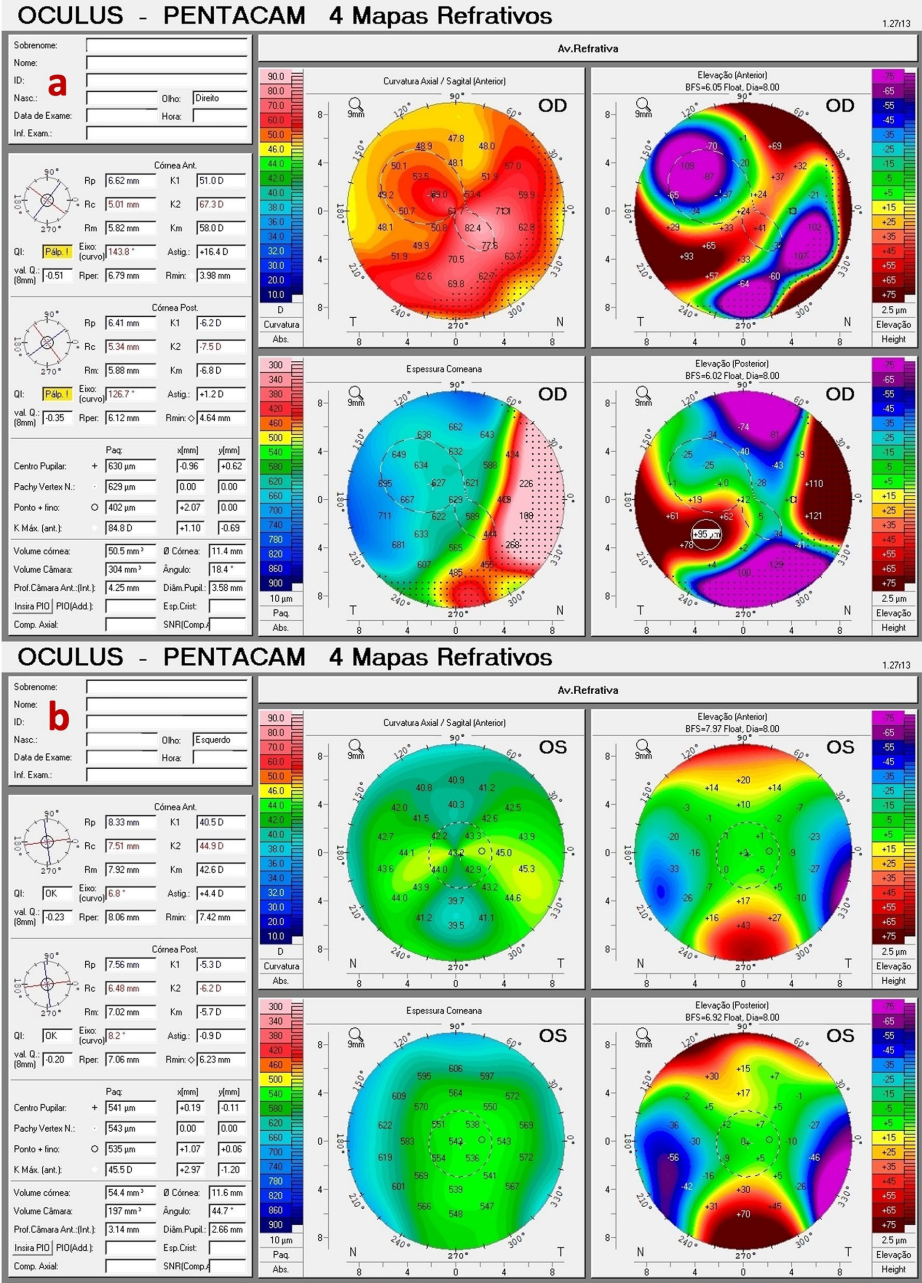
Pentacam Quad maps of a pellucid marginal degeneration (PMD) after trauma in a right eye (**a**) and subclinical PMD-like pattern in a left eye (**b**)

**Fig. 3:**
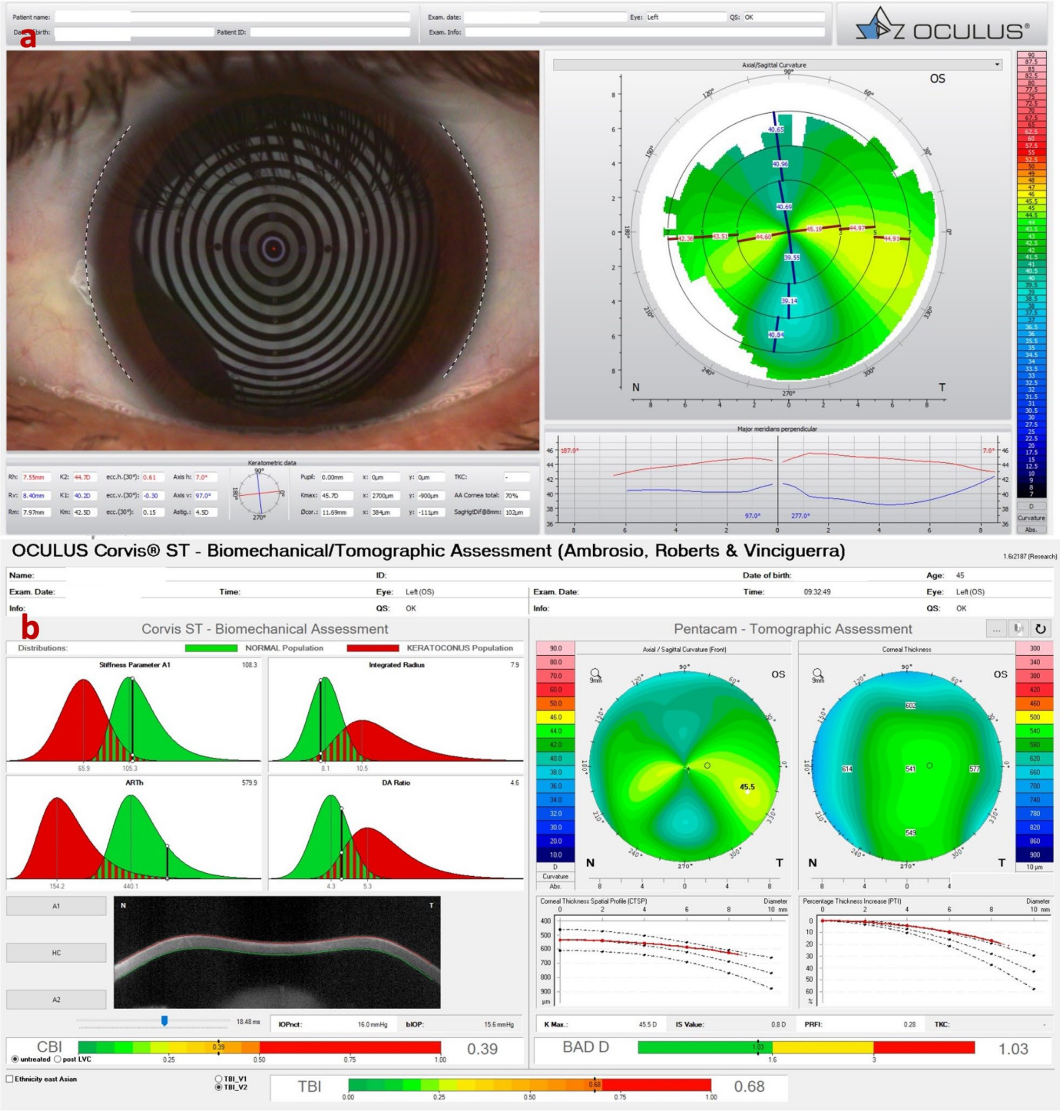
Mild, subclinical or fruste pellucid marginal corneal degeneration. **a** Keratograph 5M shows the Placido rings and the axial curvature topography of a subclinical pellucid marginal degeneration-like pattern in a left eye (OS). **b** Tomographic-Biomechanical Display shows borderline Corvis biomechanical index (CBI) and abnormal tomographic biomechanical index version 2 (TBIv2)

We report a case of a 45-year-old male with ectasia in the right eye (OD) secondary to trauma at the age of eight years. His uncorrected visual acuity (UCVA) was hand motion in the OD and 20/150 in the left eye (OS), and with DCVA, there was no improvement in OD and 20/20 in OS. The OD has a peripheral nasal and temporal thinning pattern on the OCT (Fig. 1a and b) and Scheimpflug (Fig. 1c) examinations. The peripheral thinning pattern was interpreted as a "secondary" trauma-related or aggravated PMD ectasia with peripheral thinning. Interestingly, his left eye has findings that may be related to a subclinical (fruste) or very mild PMD-like pattern (Figs. 2 and 3), with mild epithelium thinning, a Score (Anterion OCT) of 1.4 [27], and a tomographic biomechanical index (TBI) of 0.68 [17].

KC is the most common ectatic clinical condition. It is bilateral, asymmetric, and commonly a progressive ectatic corneal disease due to chronic biomechanical failure and stromal thinning [24, 28]. A study involving 1,625 Japanese patients with keratoconus and 20 patients with PMD identified 17 cases with simultaneous peripheral and central thinning patterns resembling PMD and KC. The authors concluded that PMD with or without KC might be a variant of KC or a different manifestation of the same etiologic component [29]. At the same time, the entire pathophysiology of ECD still needs to be fully understood. Nevertheless, there is an understanding of an interaction between genetic and environmental factors, as proposed by McGhee in the two-hit hypothesis [2]. The exact role of genetic predisposition and environmental factors are variable and cannot be assessed because there is no decisive genetic test for KC. There is a global agreement that while KC may present with a high degree of asymmetry, the disease is typically bilateral [23].

**Fig. 4:**
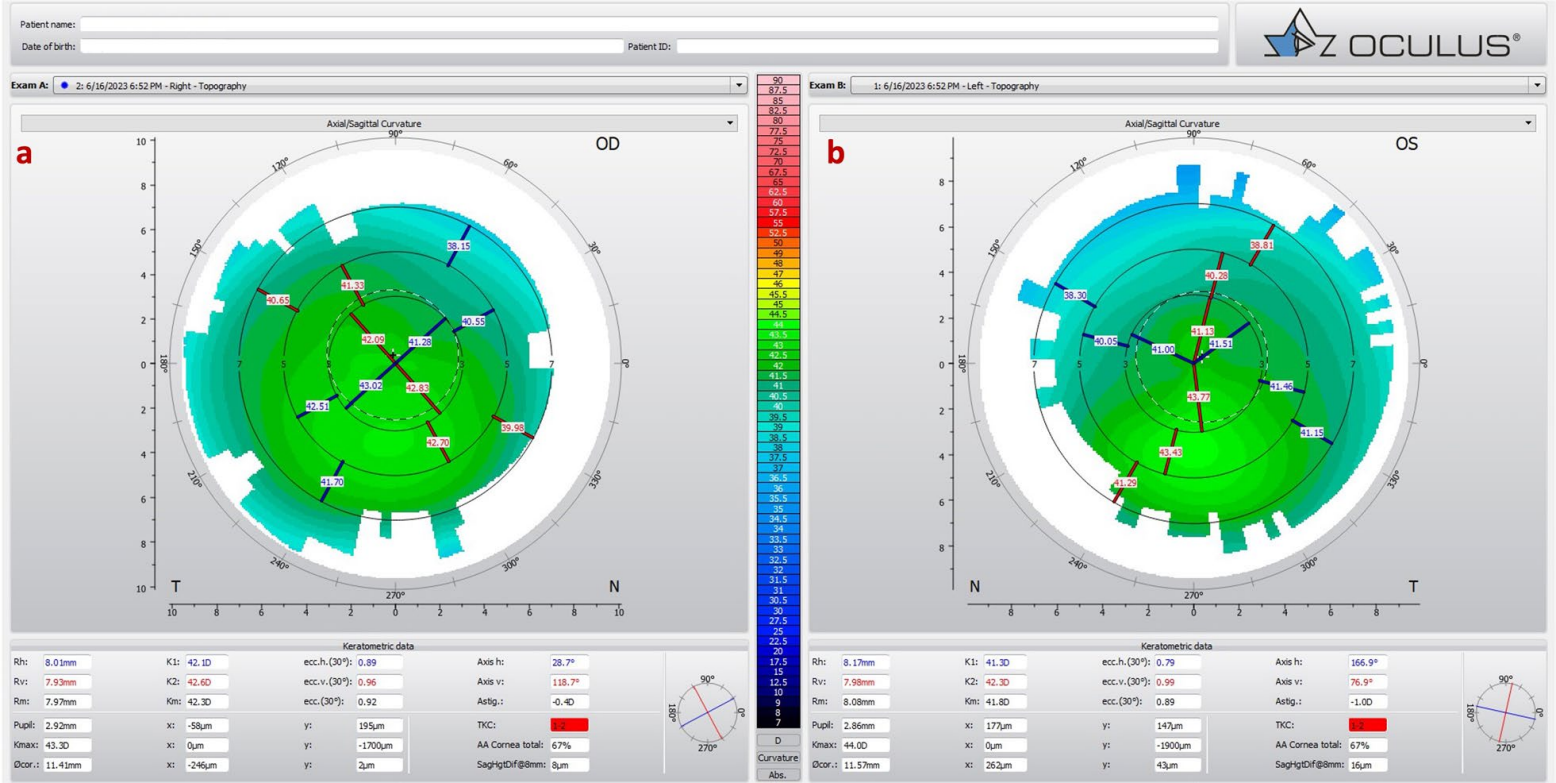
Keratograph 5M shows the topography of a low keratometry keratoconus (KC) with a maximum keratometry (Kmax) of (**a**) 43.3 D in the right eye (OD) and (**b**) 44.0 D in the left eye (OS)

**Fig. 5:**
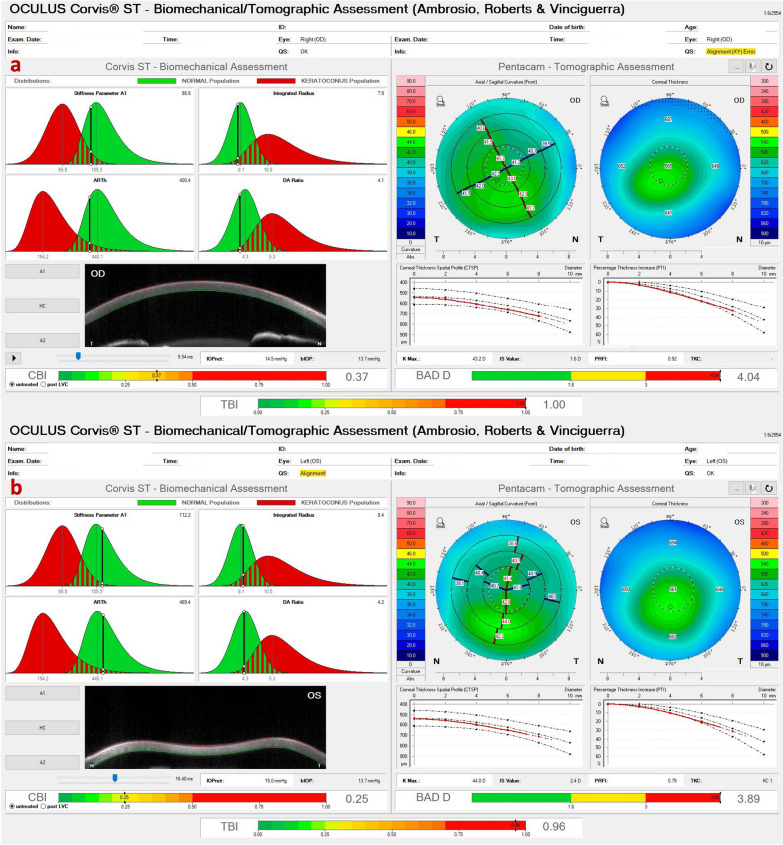
Tomographic-Biomechanical Display of the patient with low keratometry KC shows tomographic biomechanical index (TBI) of (**a**) 1.0 in the right eye (OD) and (**b**) 0.96 in the left eye (OS) and Belin/Ambrósio enhanced ectasia display deviation (BAD-D) of (**a**) 4.04 in OD and (**b**) 3.89 in OS

**Fig. 6:**
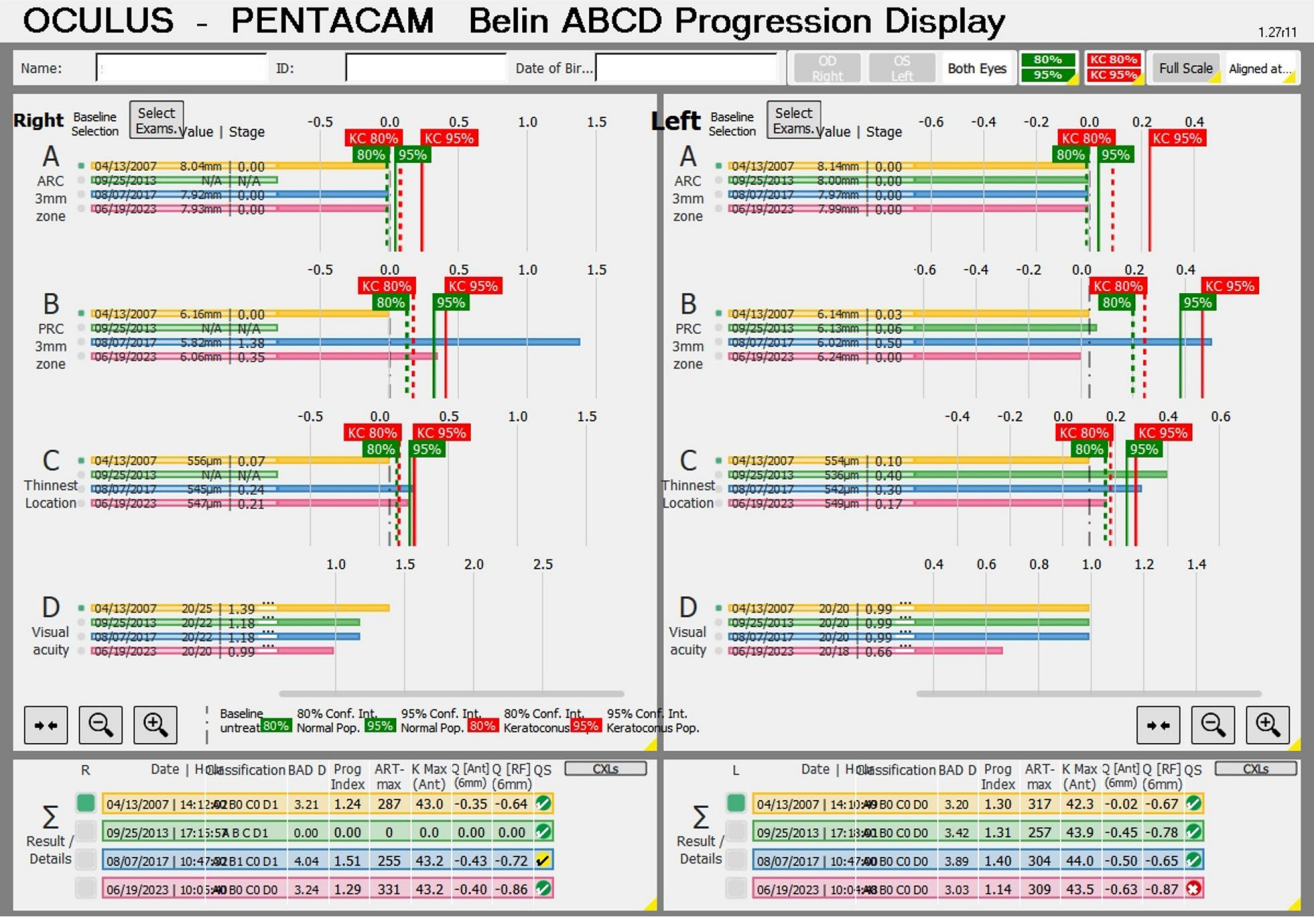
Tomographic ABCD ectasia/keratoconus (KC) staging shows the patient with low keratometry KC demonstrating stability over ten years in all parameters

Some patients present with KC despite the relatively low keratometry. Typically, such cases have a maximum keratometry (Kmax) lower than 47.6 D but present with other topometric irregularities, including inferior steepening (I-S) higher than 1.6 D and KISA higher than 60, the traditional criteria according to Rabinowitz [30]. In the case of a 65-year-old man with optimized wavefront manifest fraction [31], his DCVA was 20/20 (− 3.00/ − 0.50 × 75) in OD and 20/20 (− 2.50/ − 0.50 × 166) in OS. He presented in both eyes Placido topometric changes typical of KC with inferior steepening and relatively low keratometry (Kmax of 43.3 D in OD and 44.0 D in OS) (Fig. 4), along with tomographic and biomechanical changes (Fig. 5) but remarkable stability in the tomographic ABCD ectasia/KC staging for 16 years (Fig. 6).

Patients presenting with clinical ectasia in one eye but with the contralateral eye with normal anterior curvature (topography) and normal vision have been classically referred to as one of the possible categories of FFKC [32]. While there is no unified consensus on the definitions of keratoconus suspect (KCS) [33] and FFKC in the literature [34], such asymmetric cases have been studied using advanced imaging to demonstrate an improved ability to detect early or preclinical ECD [17, 35–39]. There is no definitive consensus, and there are currently different clinical situations potentially considered as FFKC, including the normal topographic eye of very asymmetric ectasia (VAE) cases or even a normal topographic eye that naturally evolves to clinical ectasia when longitudinally followed [12, 13, 17].

**Fig. 7:**
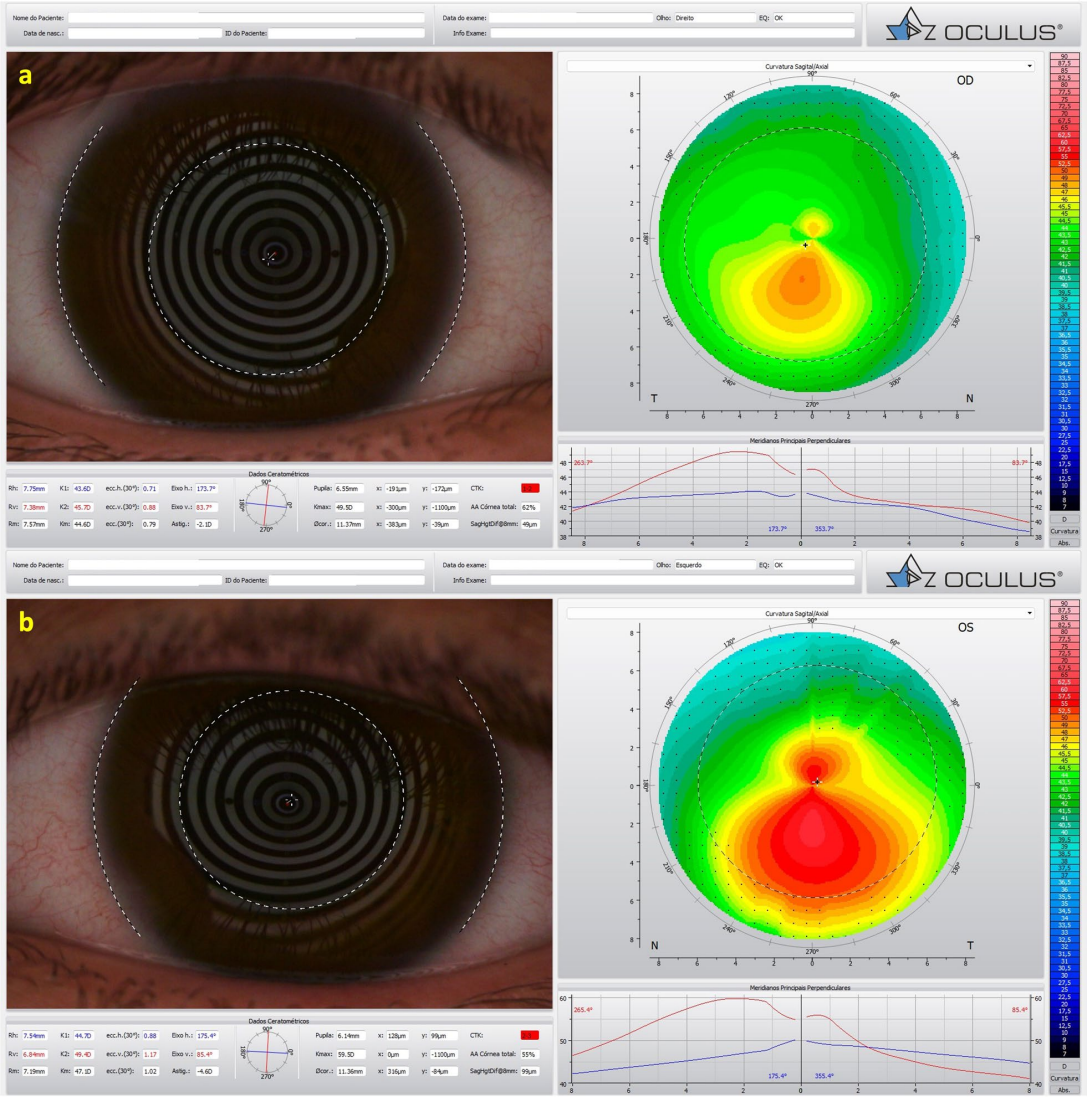
Keratograph 5M shows the Placido rings and the topography of (**a**) a very asymmetric ectasia (VAE) with a moderate keratoconus (KC) in the right eye (OD) and (**b**) advanced disease in the left eye (OS) of twin 1

**Fig. 8:**
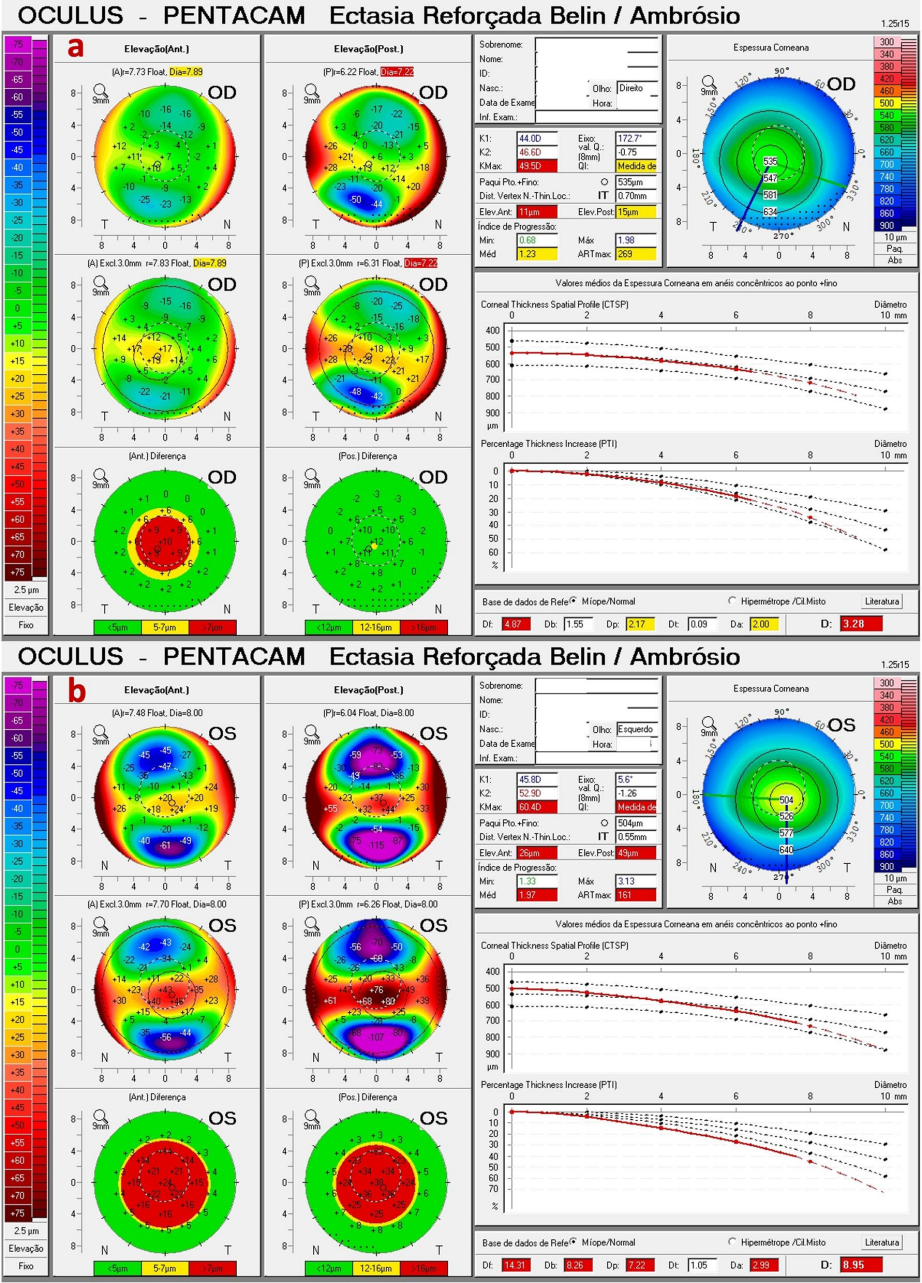
The Pentacam Belin/Ambrósio enhanced ectasia display deviation (BAD-D) shows (**a**) a value of 3.28 in the right eye (OD) and (**b**) 8.95 in the left eye (OS) of twin 1

**Fig. 9:**
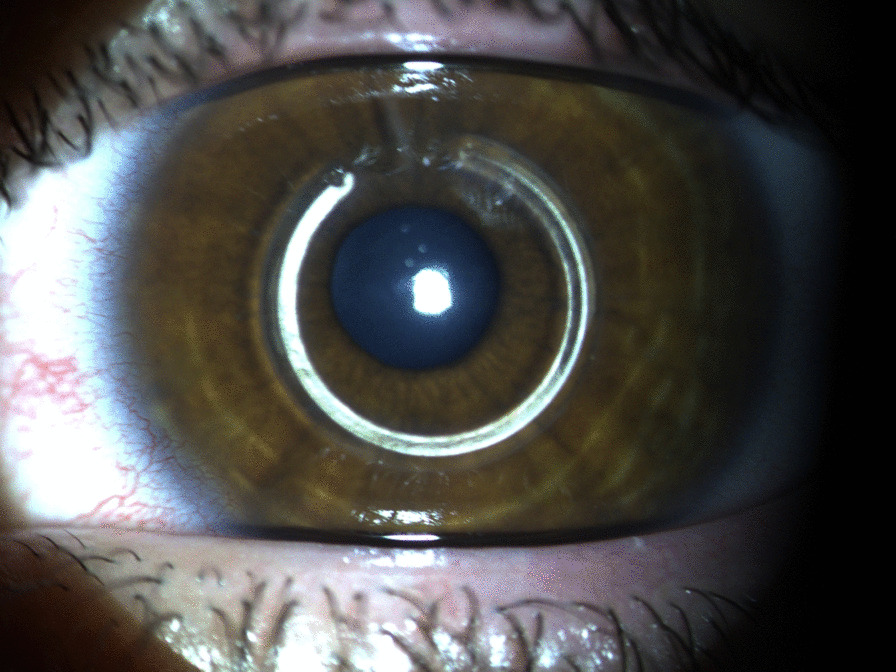
Slit-lamp biomicroscopy of intrastromal corneal ring segments (ICRS, AF 320/200) in the right eye of twin 1

**Fig. 10:**
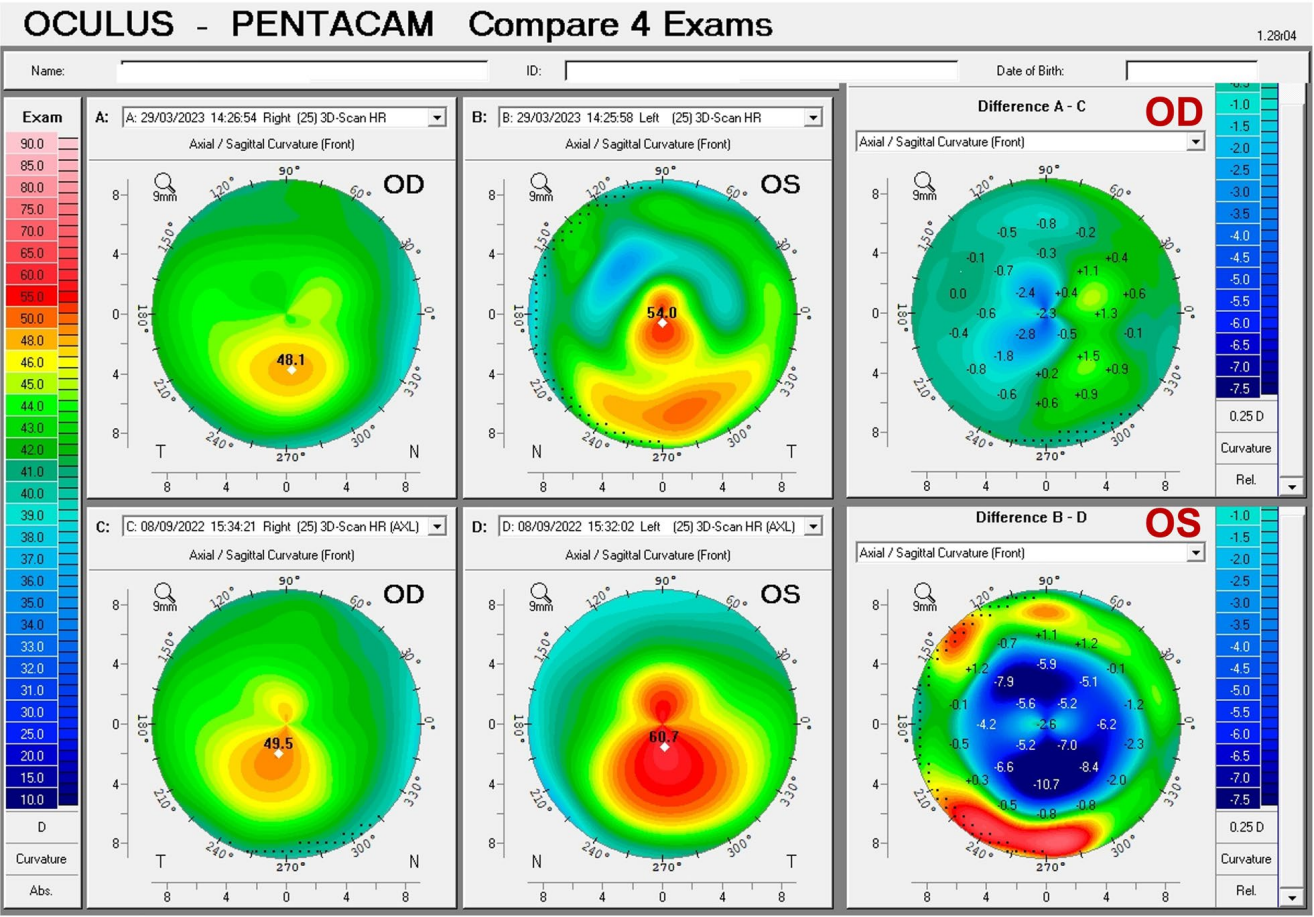
Pentacam anterior curvature differential maps shows anterior curvature maps from the right eye (OD) in March 2023 and September 2022, respectively (**a** and **c**); anterior curvature maps from the left eye (OS) in March 2023 and September 2022, respectively (**b** and **d**). Note that there was no evident progression of the ectatic disease, even a mild keratometric reduction in OD (**a**–**c**) with clinical treatment with oral supplementation of vitamin B2 and a decrease in curvature in OS (**b**–**d**) with the intrastromal corneal ring segments

We report the case of a pair of twin brothers who presented at 12 years old. They are the sons of a patient with PMD (Figs. 1, 2, and 3). Twin 1, who has an eye allergy and admits to moderate to severe eye rubbing, had VAE with mild KC in the OD, DCVA of 20/25 in OD, and more advanced disease, giving a wavefront-optimized DCVA of 20/60 in OS (Fig. 7). The Pentacam BAD also showed alterations in both eyes (Fig. 8). The proposed treatment was the implantation of a 320° arc 200 µm Ferrara Ring ICRS (Fig. 9) assisted by a femtosecond laser in the left eye as an alternative to a keratoplasty procedure [40, 41]. The right eye had conservative treatment, considering the risk of losing lines of DCVA [42]. At the same time, crosslinking should be indicated early in pediatric patients with confirmed keratoconus because of the risk of missing the opportunity to intervene. Close follow-up and patient and family education are mandatory in such situations, stressing the relevance of eye rubbing aggravating the disease [22]. A multidisciplinary approach for systemic allergies was initiated, and topical olopatadine (2.22 mg/mL) was prescribed once a day for six weeks, along with preservative-free artificial tears. Oral supplementation included omega-3 essential fatty acids and 200 mg of riboflavin (J. Jarstad, MD; personal communication 2018). Figure 10 shows the topometric follow-up after nine months, with significant flattening of more than 10.0 D after the ICRS implantation in the left eye and moderate 2.2 D flattening in the right eye. DCVA remained 20/25 OD and improved to 20/30 OS. While continuous follow-up is unquestionable, this case is an anecdotal example of the benefit of using oral riboflavin and natural sunlight exposure as a less invasive CXL technique. Interestingly, an ex vivo experiment involving sunlight exposure in porcine corneas soaked with riboflavin resulted in increased corneal stiffness [43].

**Fig. 11:**
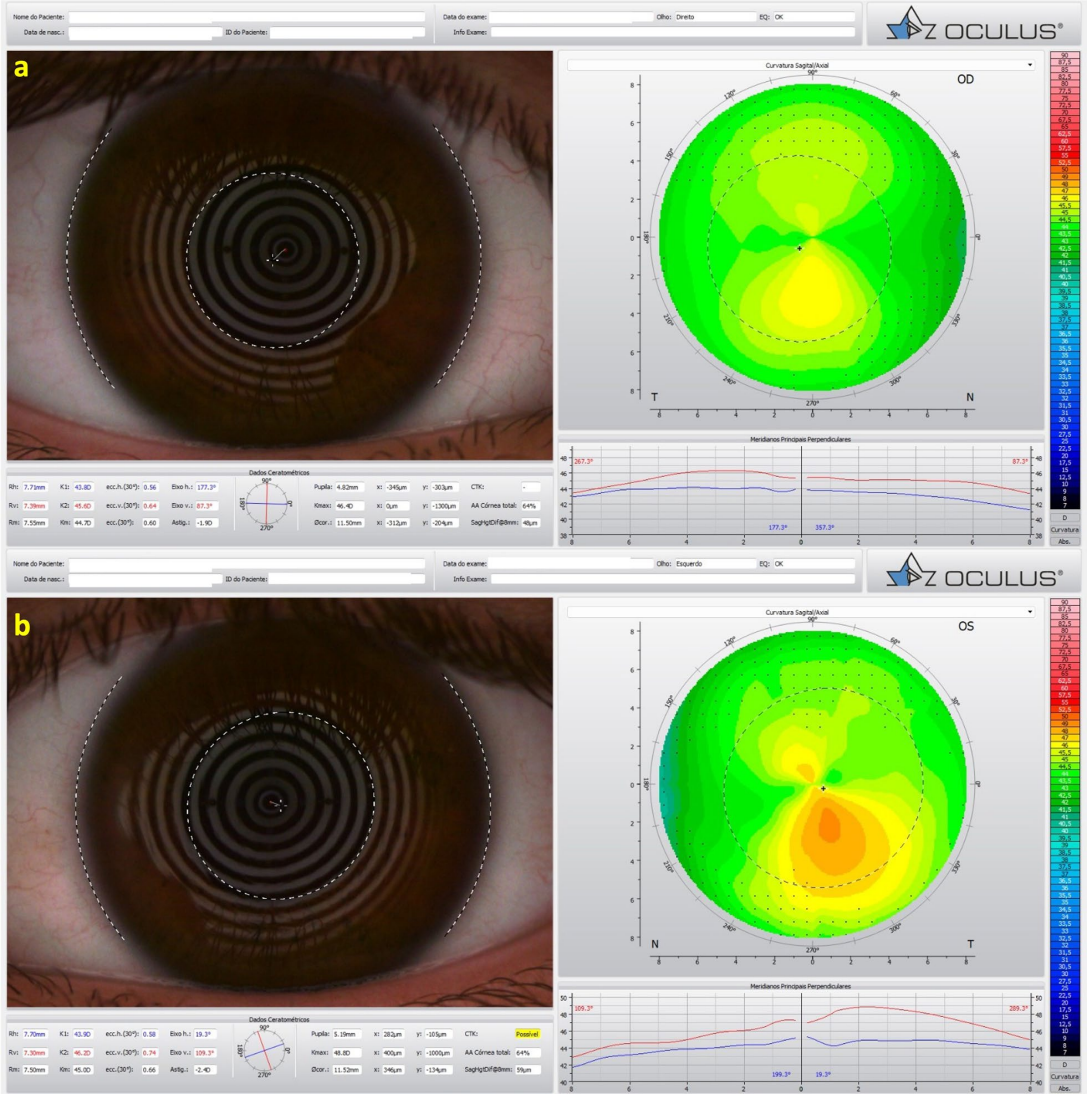
Keratograph 5M shows the Placido rings and the topography of a typical topography in the right eye (**a**) and an forme fruste keratoconus (FFKC) in the left eye (**b**) of the twin 2

**Fig. 12:**
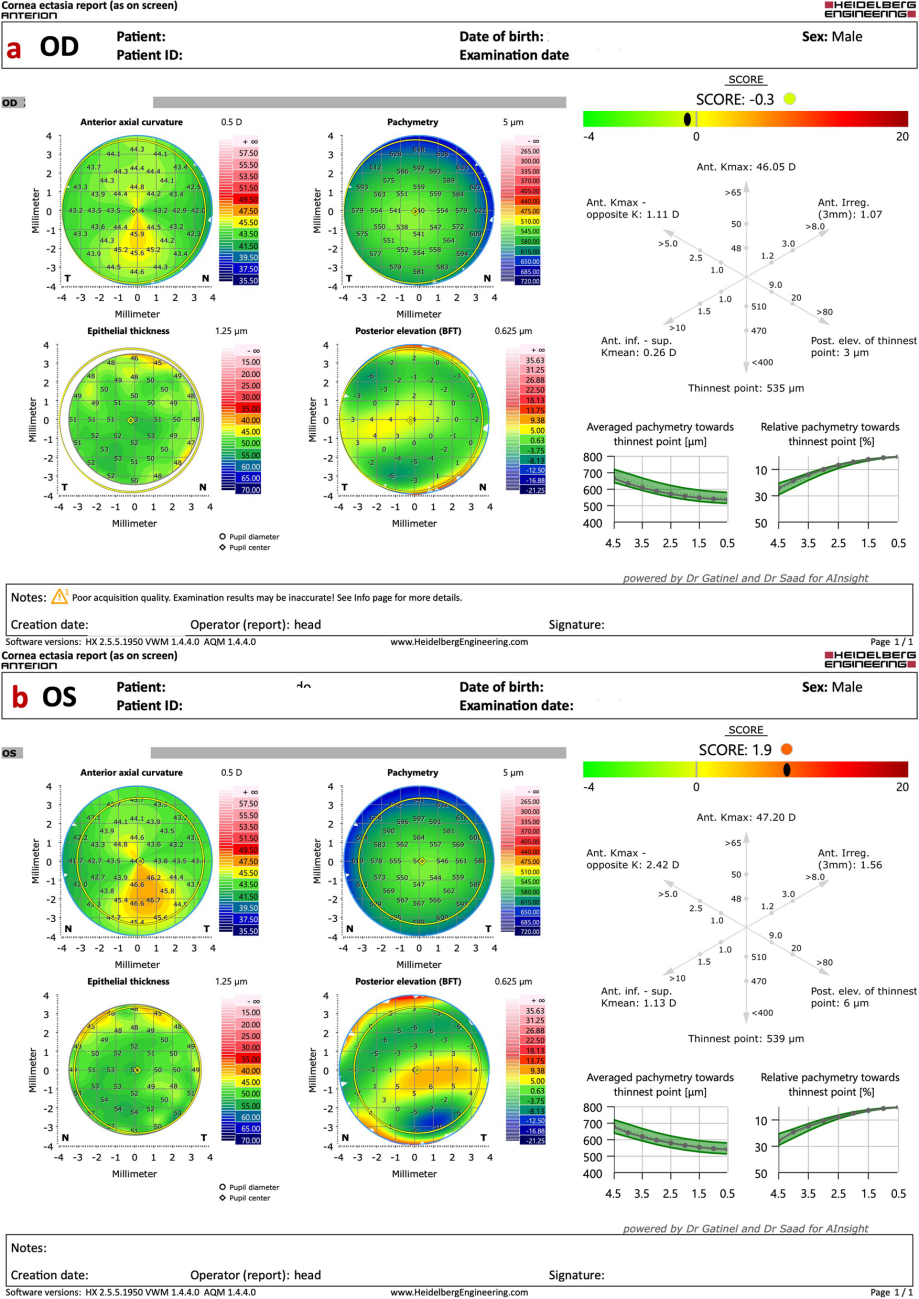
Cornea ectasia report from optical coherence tomography (OCT) of the right eye (OD) of twin 2, demonstrating a Gatinel score of − 0.3 in the OD (**a**) and an abnormal score of 1.9 in the left eye (OS) (**b**)

**Fig. 13:**
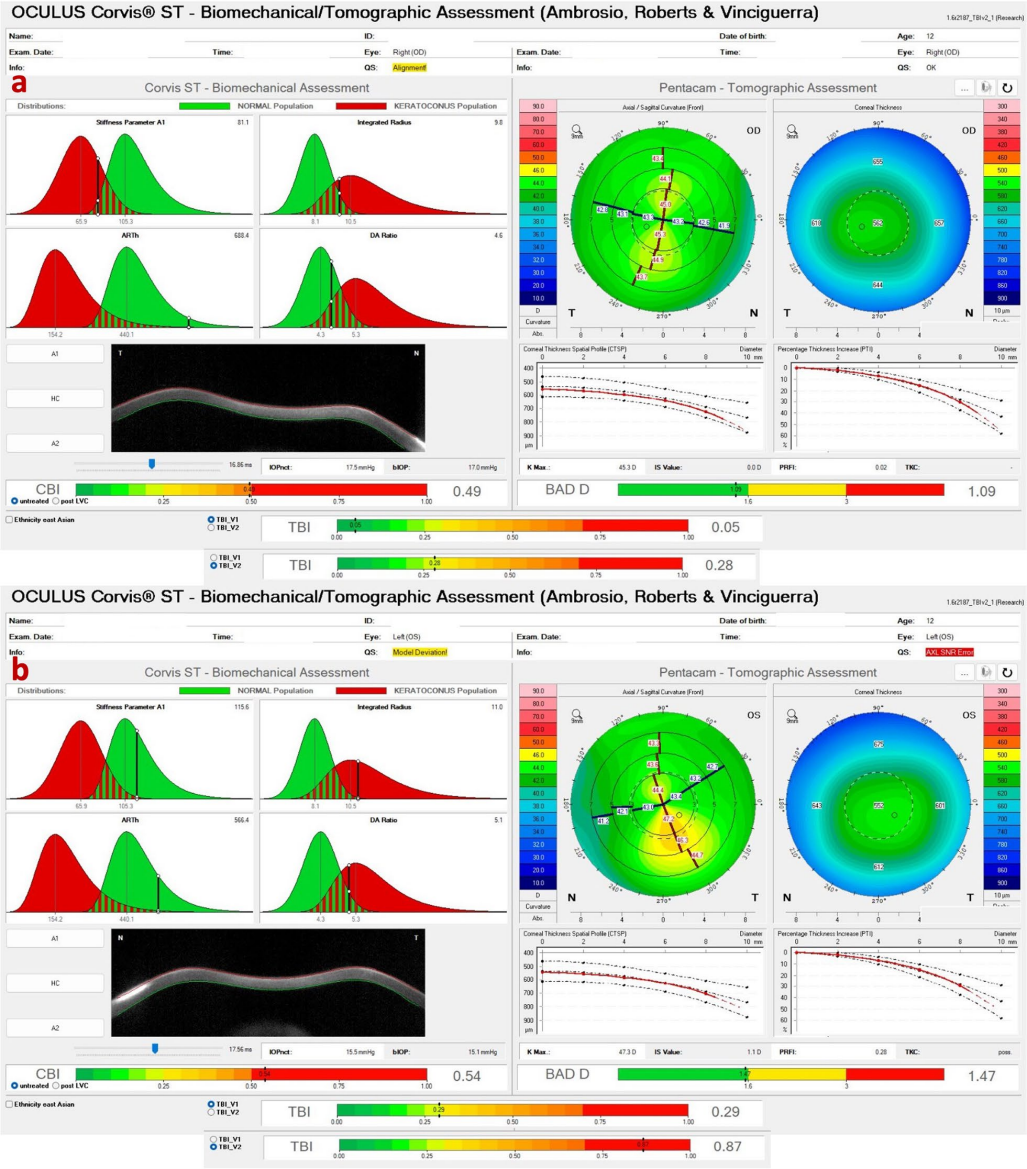
Corvis ST Tomographic-Biomechanical Display shows **a** forme fruste keratoconus (FFKC) in the right eye (OD) and (**b**) subclinical keratoconus (KC) in the left eye (OS) of twin 2. Despite a relatively regular anterior tomographic assessment (top right), note the abnormal tomographic biomechanical index version 2 (TBIv2) values of 0.28 in OD

Interestingly, identical twin 2 denied ocular itching and presented with myopic astigmatism and DCVA of 20/20 in both eyes. Nevertheless, twin 2 had clinical findings consistent with FFKC or subclinical KC in both eyes. (Table 2 and Figs. 11, 12, and 13). The Gatinel/Saad Score of 1.6 in OD and − 0.3 in the OS from the Anterion swept-source OCT (Fig. 12) and the epithelial thickness map were unremarkable in both eyes. Despite a regular tomographic assessment, we observed an abnormal Corvis biomechanical index (CBI) of 0.49 OD and 0.54 OS [18]. The optimized tomographic biomechanical index version 2 (TBIv2) values 0.28 in OD and 0.87 in OS were higher than the tomographic biomechanical index version 1 (TBIv1) (Fig. 13) [44].

**Table 2:** Summary of clinical parameters from of the father with secondary ectasia in the right eye (OD) and pellucid marginal degeneration (PMD) in the left eye (OS), and the twin brothers with very asymmetric ectasia (VAE)

Parameter	Father		Twin 1		Twin 2	
	OD	OS	OD	OS	OD	OS
Kmax (Front/Diopters)	84.8	45.5	49.5	60.4	45.3	47.4
KISA	106,614.00	219.00	52.94	221.16	3.22	5.41
I-S value (Diopters)	10.13	38	44	97	19	30
ARTmax (μm)	12	582	269	161	434	443
Pachy Min (μm)	402	535	535	504	557	552
BAD-D (v3)	9.81	1.03	3.27	8.95	1.09	1.29
SPA-1	74.2	108.3	98.5	84.9	105.3	115.6
CBI	0.99	0.39	0.77	0.98	0.49	0.54
TBIv1	0.96	0.29	0.98	1.00	0.05	0.29
TBIv2	0.88	0.68	1.00	1.00	0.28	0.87
AXL—PCI (mm)	26.42	24.92	24.29	24.60	24.93	24.84
AXL—swept-source OCT (mm)	26.45	24.98	24.26	24.39	25.10	24.94
Anterion SCORE*	118.3	1.4	4.4	21.7	− 0.3	1.9

*Kmax =* maximum keratometry; *I-S value =* inferior-superior asymmetry at 6mm diameter in axial diopters; *ARTmax =* Ambrósio´s relational thickness in the meridian with maximal (more abrupt) progression; *Pachy Min =* thinnest (minimal) pachymetry; *BAD-D (v3) =* Belin/Ambrósio enhanced ectasia deviation (third version); *SPA-1 =* stiffness parameter at the first applanation; *CBI =* Corvis corneal biomechanical index; *TBIv1 =* tomographic biomechanical index version 1; *TBIv2 =* tomographic biomechanical index version 2; *AXL =* axial length; *PCI =* partial coherence interferometry

^*^Score after intracorneal stromal ring implantation (pre-op not available)

### Prognostic information

Patients and their families must understand that surgery in ECDs, particularly in KC, is therapeutic and not a refractive procedure. Then, the primary purpose of the surgical treatment is to restore vision, not to reduce or eliminate the need for vision correction, as in elective refractive surgery [5]. The paradigm shift defined by Seiler when he published his results describing the halting of progression in KC crosslinking and ICRS that could be utilized earlier in the disease process than penetrating keratoplasty came through a paradox as well [2, 3].

The paradigm shift relates to the fact that, formerly, visual rehabilitation was the central premise of KC management. Nevertheless, the latest treatment modes aim to prevent visual loss before it happens. Conversely, no surgery should be indicated if not needed, such as ECDs with good vision with glasses with no signs of progression. However, it must be correctly suggested and performed to avoid losing the opportunity for better outcomes if vision deteriorates or ECD advances [3]. Lindstrom and coworkers proposed that the optimal economic impact of crosslinking emerges when performed at an earlier stage of the disease and a younger age, leading to enhanced work productivity, reduced costs, and an elevated quality of life [45]. The proposed treatment for twin 1 was the implantation of ICRS in the OD due to moderate KC (Fig. 9). In OS, we opted for clinical management, including ocular allergy control with guidance not to rub or scratch the eyes and to supplement 200 mg of vitamin B2 and natural sunlight exposure, a noninvasive crosslinking method [43]. Figure 10 shows the topometric follow-up over nine months with mild but significant corneal flattening.

### Characterizing ectasia susceptibility

Progressive corneal ectasia can be iatrogenic after different types of corneal refractive surgery, similar to LASIK in patients with changed biomechanical properties, known as FFKC. After refractive laser correction, FFKC was recognized as the main factor for developing progressive ectasia [7, 11]. Unilateral ectasia has also been described in patients who underwent monocular refractive surgical procedures, which remain stable in the unoperated fellow eye [16]. Nevertheless, unilateral post-LASIK ectasia has been reported, with the onset of ectasia ranging from four months to 18 years [46].

**Fig. 14:**
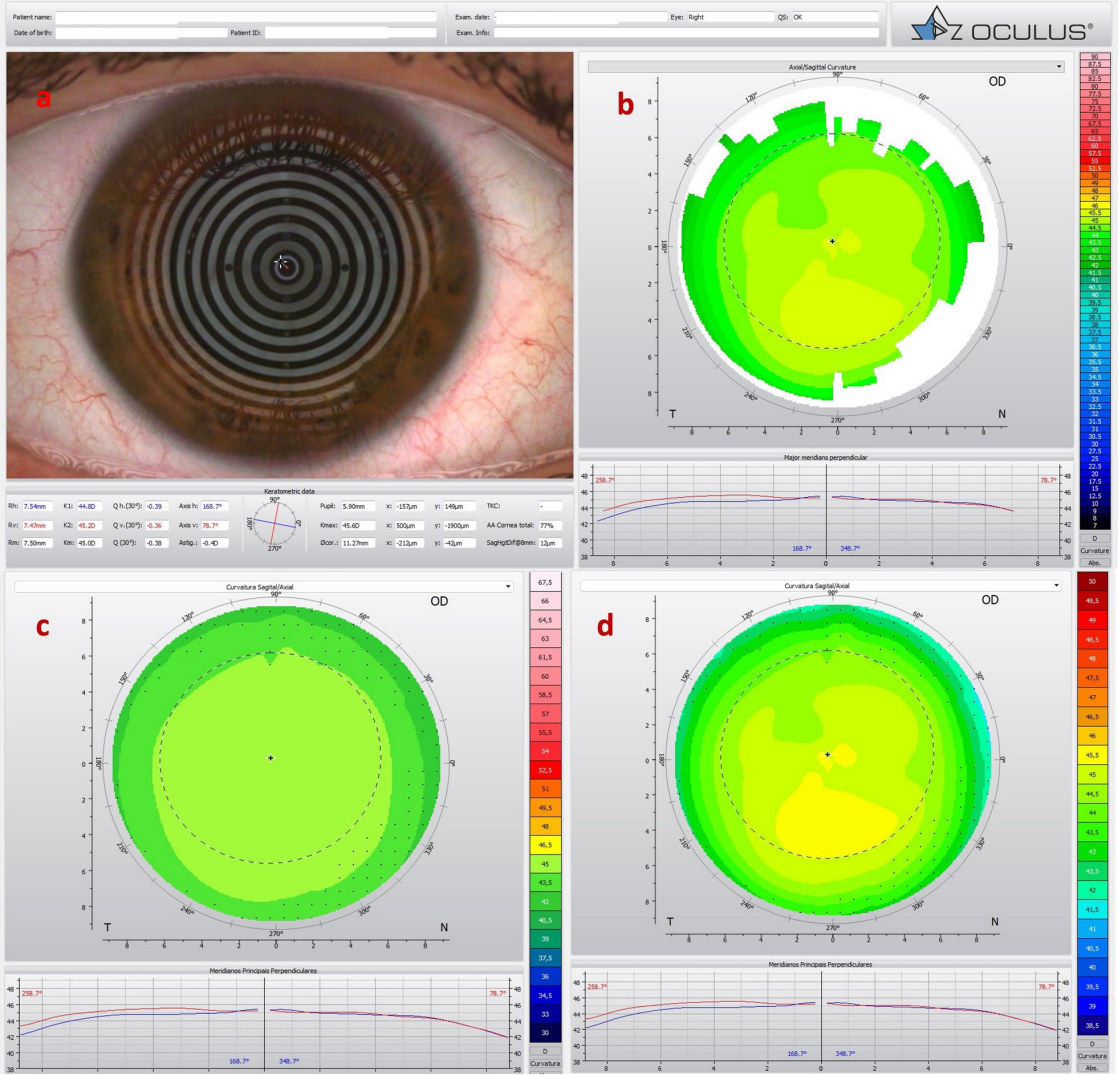
Keratograph 5M shows the unoperated right eye (OD) of a patient that developed ectasia in the contralateral left eye (OS) after unilateral laser in situ keratomileusis (LASIK). **a** The Placido rings; **b**–**d** The axial curvature topography with the Ambrósio-2 absolute scale (**b**), Klyce/Smolek absolute 1.5 D scale (**c**) and the absolute 0.5 D Atlas scale (**d**)

**Fig. 15:**
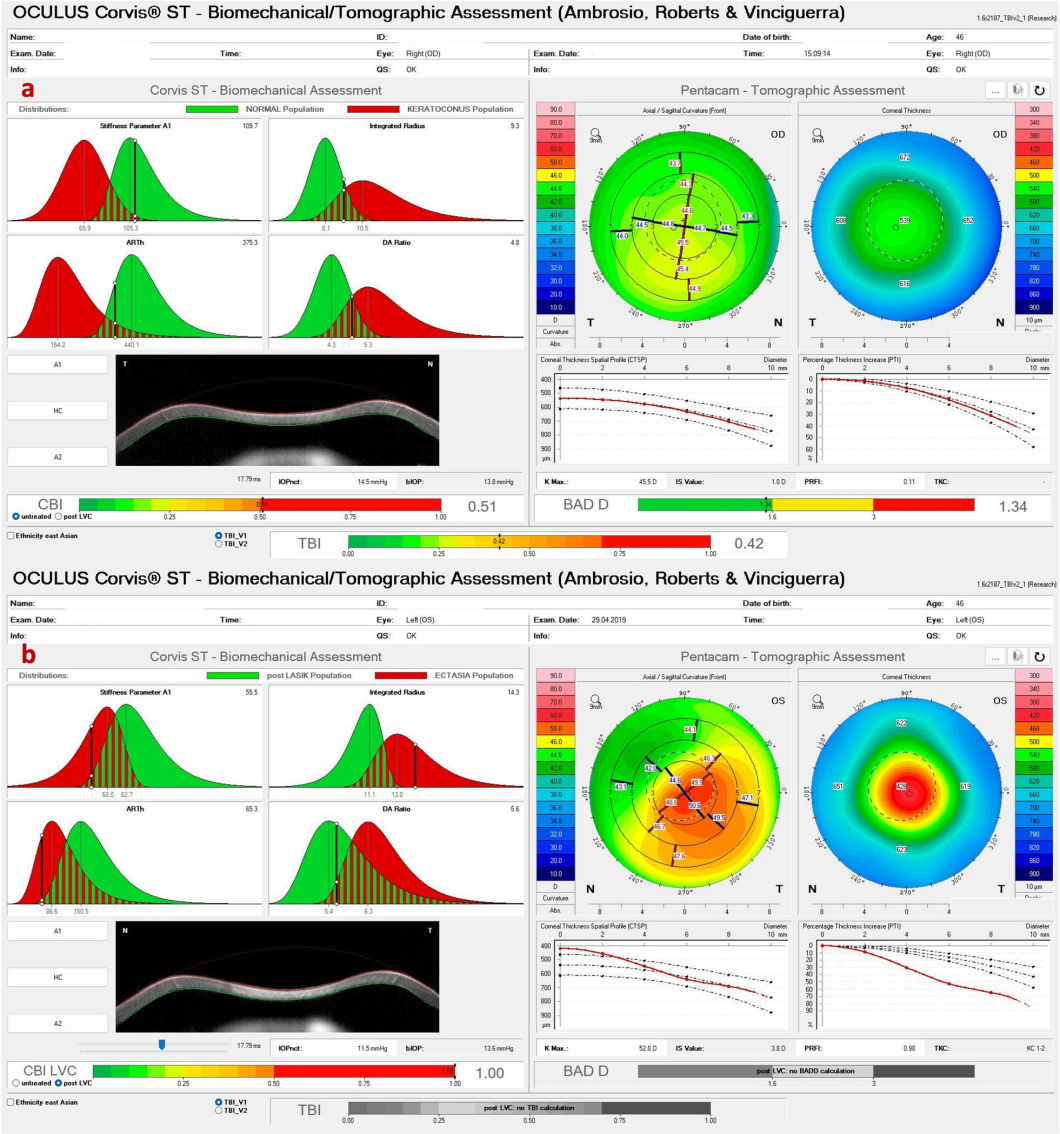
Corvis ST Tomographic-Biomechanical Display of the same patient of Fig. 14 shows (**a**) abnormal Corvis biomechanical index (CBI, 0.51) and tomographic biomechanical index (TBI, 0.49) despite borderline BAD-D (v3) 1.34 in the unoperated right eye (OD) and (**b**) high CBI post-LVC in the left eye (OS) with post-LASIK ectasia. BAD-D (v3), Belin/Ambrósio enhanced ectasia deviation (third version); LVC, laser vision correction; LASIK, laser in situ keratomileusis

**Fig. 16:**
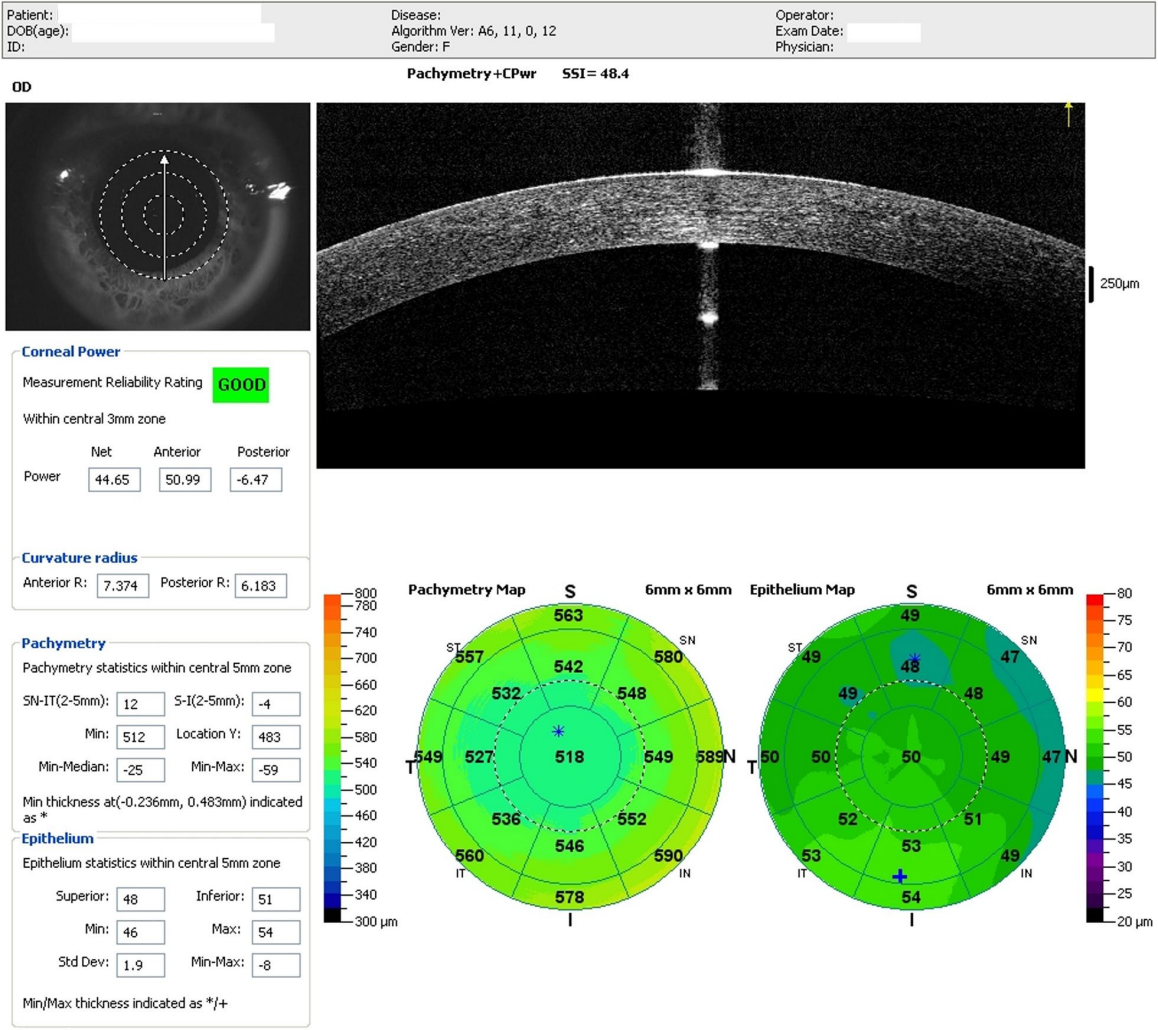
Optical coherence tomography (OCT) of the cornea shows the epithelial map and pachymetry of the contralateral eye (the right eye) of the same patient in Figs. 14 and 15. Note mild but relevant epithelial thinning

We reported a case of unilateral progressive corneal ectasia after unilateral LASIK for myopic astigmatism in a 31-year-old woman with no identifiable preoperative risk factors for ectasia [16]. She had uncomplicated LASIK in OS and noted progressive deterioration of vision one year after surgery. The preoperative Placido disk-based topography revealed regular and symmetric bowtie with-the-rule astigmatism in both eyes without signs of ECD (Fig. 14). The advanced tomographic and biomechanical evaluation demonstrates a high TBI in the unoperated right eye of 0.42 and a high CBI post-LVC in the left eye that developed post-LASIK ectasia (Fig. 15). The epithelium evaluation with OCT also enhanced the diagnosis of a higher risk of progressive iatrogenic ectasia after LVC, as shown in the OD of this patient (Fig. 16).

The assessment of ectasia risk among elective refractive surgery candidates has evolved to the characterization of the inherent susceptibility of the cornea for biomechanical decompensation and ectasia progression, which lies beyond detecting mild cases with ECD [7, 14, 47]. In addition, the ectasia risk assessment should also include the impact of the LVC procedure, which is supported by studies involving finite element analysis [48, 49]. This concept is in agreement with McGhee's two-hit hypothesis that genetic (intrinsic) and environmental (extrinsic) causes play a role in the etiology of KC [2], and the biomechanical cycle of decompensation of corneal ectasia proposed by Dupps and Roberts [28]. The current concept is that the pathophysiology of ECD is associated with a primary biomechanical abnormality: architecture and morphology instability secondary events [7, 23, 28]. Furthermore, newer treatment modalities for ECD emphasize the relevance of recognizing mild or subclinical ectatic disease in addition to ectasia risk assessment before corneal LVC [13].

## Keratoconus associated with other corneal dystrophies

KC may be associated with other corneal diseases, including posterior polymorphous corneal dystrophy (PPCD) [50, 51], and most commonly, Fuchs dystrophy [52]. These corneal disease associations emphasize the need for a multimodal diagnostic approach when evaluating patients with suspected corneal ectasia. Coexisting corneal diseases can significantly impact management strategies and treatment outcomes.

**Fig. 17:**
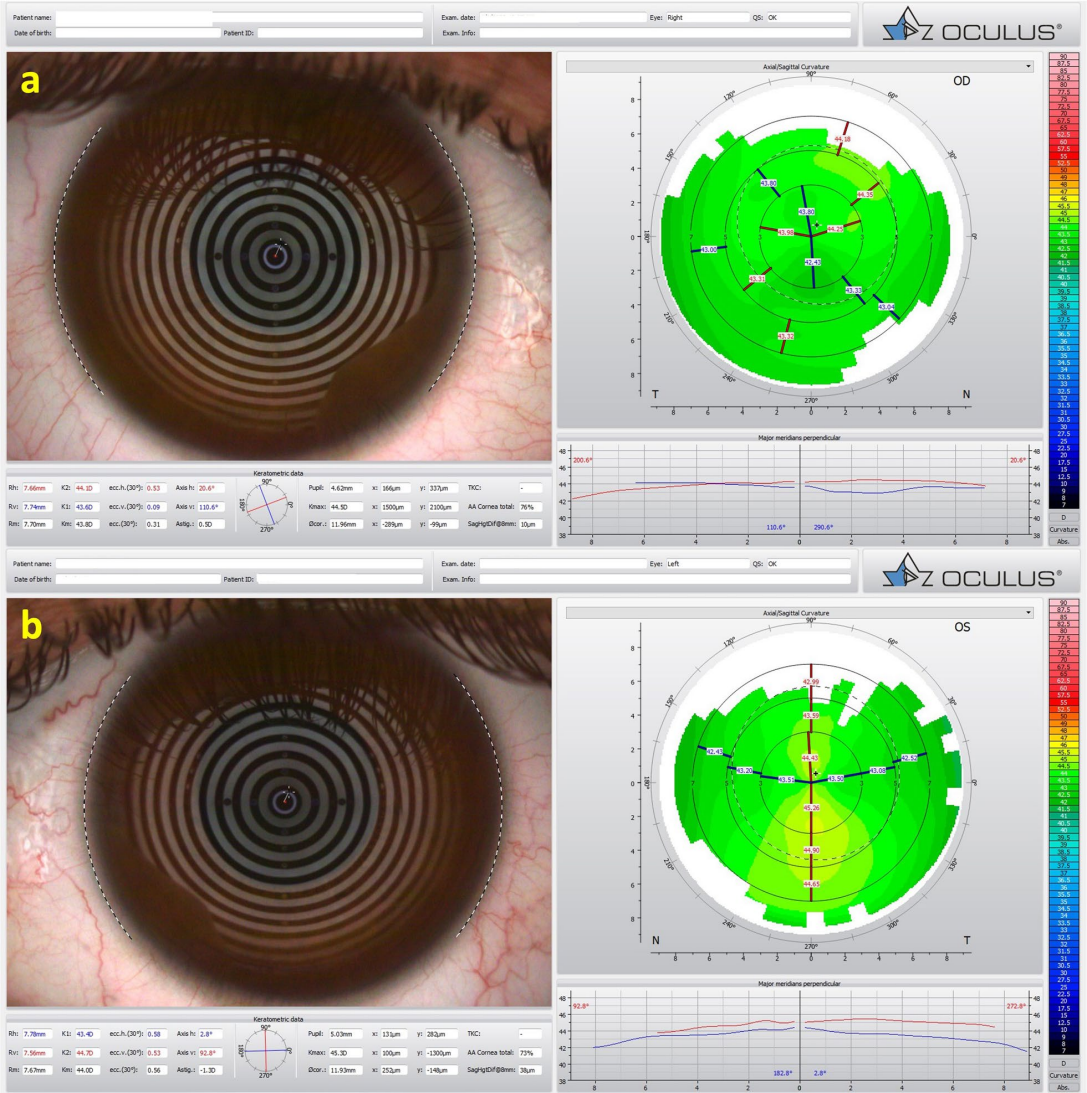
Keratograph 5M shows the Placido rings and the relatively normal axial curvature topography of a 27-year-old female with a posterior polymorphous corneal dystrophy (PPCD) and considered with (**a**) forme fruste keratoconus (FFKC) in the right eye (OD) and (**b**) a mild inferior steepening in the left eye (OS)

**Fig. 18:**
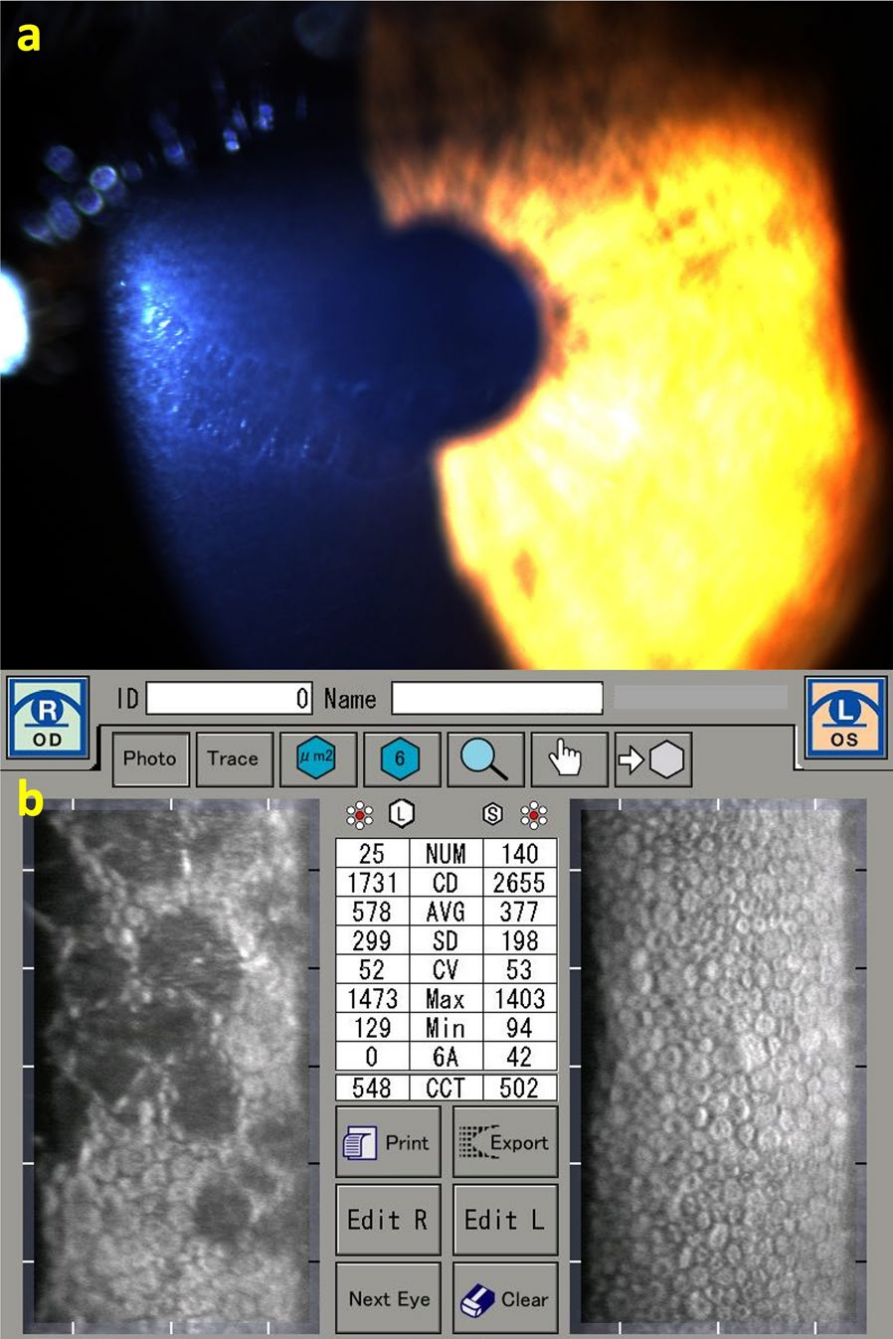
Slit-lamp biomicroscopy of the right eye (OD) with the “snail track” sign (**a**) and specular microscopy with endothelial alterations in OD (**b**)

**Fig. 19:**
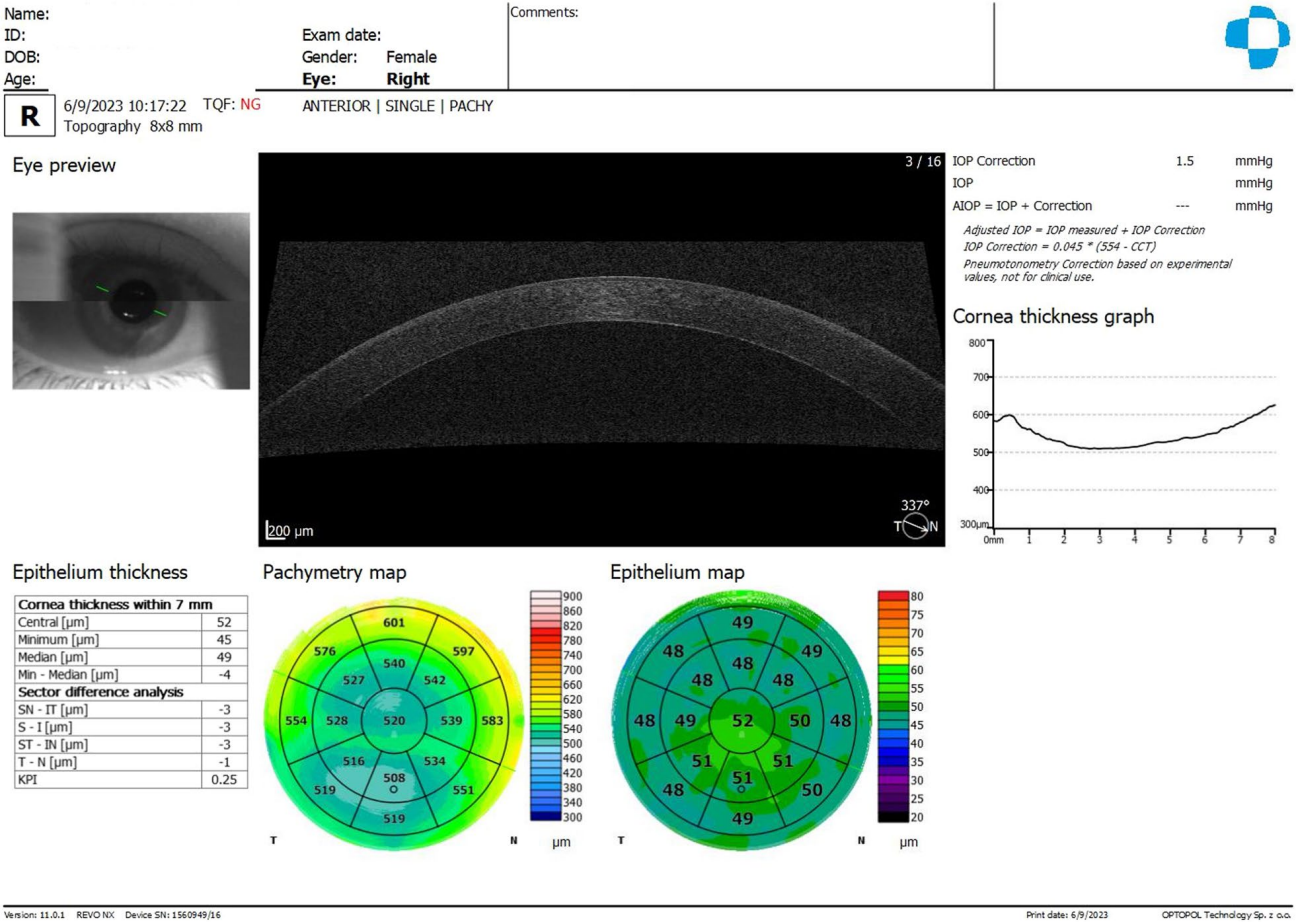
The optical coherence tomography (OCT) of the right eye shows the pachymetry and epithelium maps

**Fig. 20:**
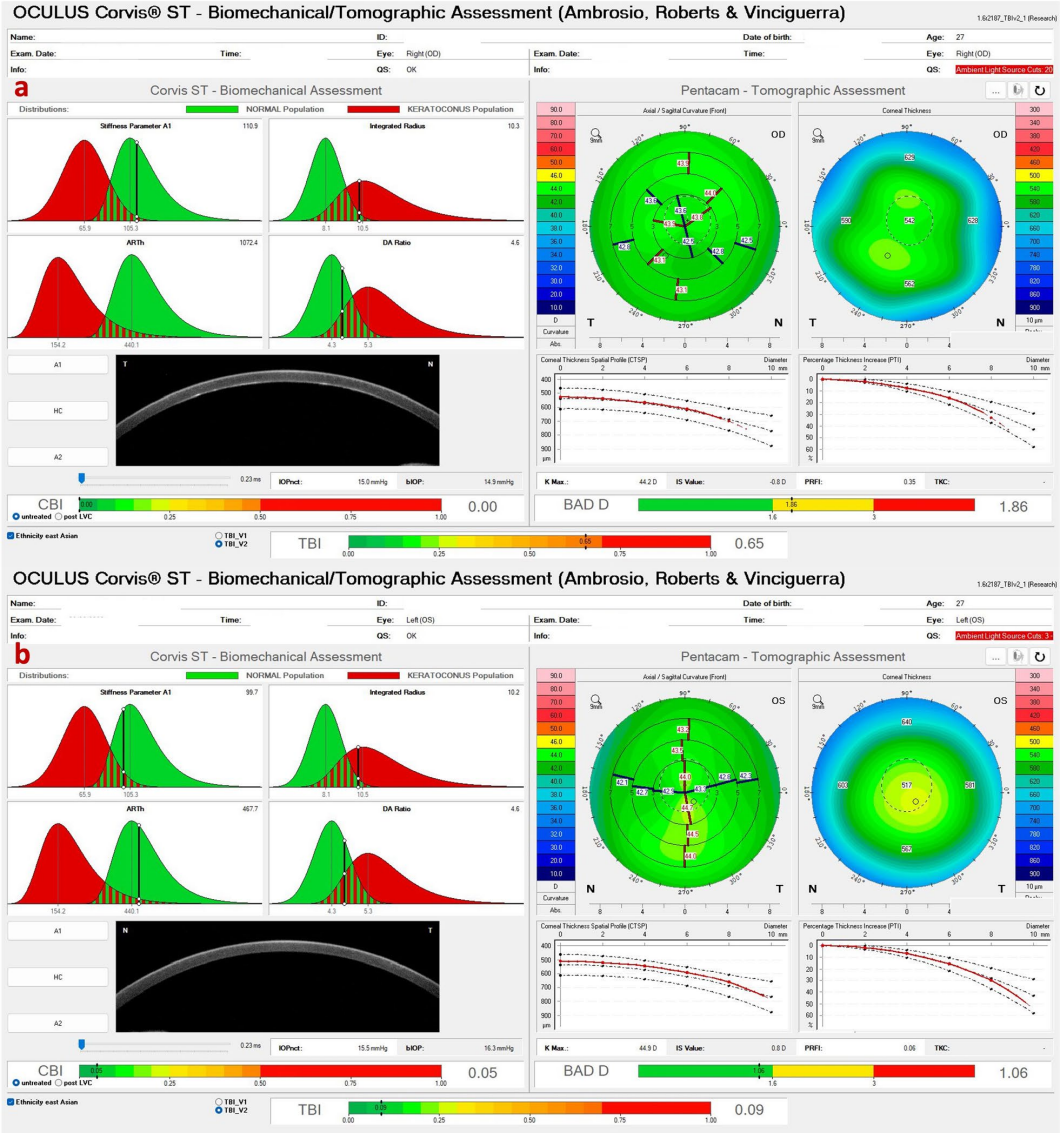
Tomographic-Biomechanical Display of the patient with posterior polymorphous corneal dystrophy (PPCD) and forme fruste keratoconus (FFKC) with (**a**) relatively high tomographic biomechanical index (TBI) in the right eye (OD) and (**b**) relatively normal TBI in the left eye (OS)

We report a case of a 27-year-old female with a PPCD and FFKC in OD and a relatively normal left eye (Fig. 17). Her DCVA was 20/25 (− 5.75/ − 0.75 × 160) in OD and 20/20 (− 6.25/ − 1.50 × 5) in OS, setting up an astigmatic anisometropia. In the slit-lamp biomicroscopy, we observe the "snail track" sign and specular microscopy with endothelial alterations in OD and a normal endothelium OS (Fig. 18). In the OCT, we can also see the corneal endothelium changes in OD (Fig. 19). The TBIv2 is high in the right eye (0.65) and within the normal range (0.09) in OS (Fig. 20). Such findings demonstrate the need for further developments, which should consider genetic factors for further elucidating the associations of corneal dystrophies.

## The quest for multimodal imaging

Different diagnostic tools are available for refractive characterization, along with corneal and anterior segment imaging. Integrating the diverse information each technology offers enables conscious clinical decisions [6]. Placido disk-based corneal topography does augment our ability to identify mild ectasia irregularities in patients with unremarkable biomicroscopy [53, 54]. Thereafter, the introduction of anterior segment tomography with 3D cornea reconstruction presented additional detail about corneal architecture with various quantitative indices resulting from the front and back (posterior) elevation and pachymetric maps [55–57].

The need to go beyond corneal shape assessment to define ectasia risk within the biomechanical field has been sustained and supported. The current concept for ectasia development is that a focal weakening in corneal structure starts a chronic cycle of biomechanical decompensation, leading to localized thinning and steepening, which clinically define ectasia progression [28]. The use of multimodal corneal imaging is also essential for the screening for ectasia risk before LVC, not only to identify cases with mild ectasia, such as VAE and FFKC but to identify cases of elevated susceptibility for biomechanical failure and ectasia after LVC [58]. The current acceptance is that the combination of the biomechanical decompensation of the stroma, which is correlated to either the impact of the method on the corneal structure or the individual biomechanical properties preoperatively determines stability or ectasia progression after LVC [16, 59, 60]. Table 2 shows the summary of clinical parameters from Scheimpflug-based corneal tomography and biomechanical analysis of the identical twins and their father mentioned above. The relevance of multimodal assessment for ECD goes beyond corneal LVC procedures, being also essential for refractive cataract surgery. Detecting corneal ectasia, even in its subtle or mild manifestations, will influence the power calculation and selection of intraocular lenses, postoperative quality of vision, and, if necessary, the accuracy and safety of fine-tuning LVC enhancements.

### Corneal topography

Placido-disk-based corneal topography characterizes the anterior or front surface of the cornea using quantitative data to generate color-coded maps [61]. It has proven to be sensitive for the detection of ectatic disease even before any loss of best-corrected visual acuity and any remarkable slit-lamp exam findings develop [53, 54].

Different topographical indices have been proposed for detecting KC [61]. While often lacking specificity, such data have proved sensitive to recognizing mild ectatic patterns (Fig. 11) [53, 54]. Therefore, this ability has historically positioned corneal topography as a mandatory exam in the screening process of refractive surgery candidates [12]. Randleman and coworkers established the Ectasia Risk Scoring System with corneal topography, pachymetric, and clinical variables [10, 62]. However, the drawbacks of limiting the analysis to the anterior curvature were pointed out when considering cases that developed post-refractive surgery keratectasia after LASIK [47, 63, 64], small incision lenticular extraction (SMILE) [65, 66], surface ablation [67], despite a relatively normal anterior shape (Fig. 14) [16].

### Corneal tomography

The Orbscan (Bausch & Lomb; Rochester, US) 3D slit-scanning system was introduced as the first corneal tomography method. Studies have found good sensitivity and specificity of Orbscan-derived parameters to discriminate early forms of KC, even in cases undetected by Placido-based topography alone [36]. AI technology was used to generate a Screening Corneal Objective Risk of Ectasia (SCORE) system, which objectively classifies the topographic map as positive or negative for the risk of developing ectasia [27, 68, 69]. Scheimpflug imaging is one of the most popular corneal and anterior segment tomography methods [70]. The Galilei Dual-Scheimpflug Analyzer (Ziemer; Port, Switzerland) combines a dual Scheimpflug camera with a Placido-disk topography system to generate 3D images of the cornea and anterior chamber. In different studies, KC indices derived from this device have been used to discriminate normal efficiently and KC eyes [71].

The Pentacam (Oculus, Wetzlar, Germany) has a rotating Scheimpflug camera and a frontal view lighting system to recreate topographic images of the cornea and anterior segment. The Pentacam Belin/Ambrósio Enhanced Ectasia Display (BAD) identifies the deviation from normality to disease (D values), facilitating clinical diagnosis of KC and ECD [37, 70, 72]. A final 'D' value is intended based on linear regression analysis (Fig. 8) [60, 70, 73]. The Pentacam random forest index (PRFI) development confirmed that AI enables enhanced analysis of Scheimpflug tomography for improving accuracy in ectasia detection [74]. A second parameter was developed with multiple logistic regression analysis (MLRA) as the boosted ectasia susceptibility tomography index (BESTi) [75], indicating that AI could further enhance the accuracy of identifying mild forms of ectasia and higher susceptibility to developing such complications [36, 37, 57, 72, 76].

### Layered or segmental corneal tomography

Further advances in corneal tomography allowed the development of layered or segmental characterization of individual corneal layers, such as the epithelium and Bowman's layer. Segmental tomography with epithelial thickness was first introduced with (VHF-US) by Reinstein and coworkers [77–79]. However, spectral-domain and swept-source OCT made it conceivable and popularized [56, 70]. Corneal epithelial indices for detecting KC have been developed with this technology, and studies propose this approach as a valuable tool in identifying milder forms of the disease [79, 80]. Figure 12 shows a Gatinel score change of 1.6 in OD and − 0.3 in the OS in the cornea ectasia report of twin 2. In addition, the OCT image with epithelium evaluation also enhanced the diagnosis of a higher risk of progressive iatrogenic ectasia after LVC (Fig. 16).

Huang and collaborators used OCT technology to establish segmentation, developing an analogous method with a comprehensive epithelial thickness map and various indices to detect KC early [81, 82]. Another study investigated the irregular Bowman's layer in normal and ectatic corneas and suggested a new Bowman's roughness index. This index had good performance in identifying KC, and when used with the Belin/Ambrósio enhanced ectasia display deviation (BAD-D) and epithelial thickness data, improved the sensitivity for identifying mild forms of ectasia [83], which can prove to be useful and increase our sensitivity to detect early stages of ectatic diseases [81]. A recent study found that OCT topography of Bowman's layer combined with AI significantly improved the detection of FFKC eyes [84].

### Corneal biomechanics

Corneal biomechanics is an essential topic for research and development in ophthalmology because of its many potential applications [28, 57]. It is presumed that in KC and other ECD, the curvature, elevation, and pachymetry changes, which remain the focus of the clinical investigation, are most probably secondary to a focal weakening that initiates a biomechanical decompensation [28, 85]. Thus, early identification of an eventual biomechanical failure beyond corneal shape analysis might enhance the sensitivity to detect milder forms of ECD.

Additionally, biomechanical analysis has become noteworthy in pre-operatory of LVC to recognize patients at higher risk of developing iatrogenic ectasia after LVC, increasing the expectedness and effectiveness of these elective procedures [17, 86, 87]. Two systems can measure the corneal biomechanical response: the ocular response analyzer (ORA; Reichert, Buffalo, NY, USA) and the Corvis ST (Oculus, Wetzlar, Germany). The AI algorithms confirmed that the arrangement of deformation parameters improved the accuracy of distinguishing healthy and KC eyes, even in mild stages [88].

In 2014, two principal parameters were developed by a multicentric international investigation group for improving corneal ectasia recognition, the CBI and the TBI [89, 90]. Vinciguerra and coworkers verified that in the training set of cases, the 0.5 criteria for the CBI correctly identified 98.2% of KC cases with 100% specificity and sensitivity of 94.1%, giving an area under the curve (AUC) of 0.983. Later, the same cutoff value in the validation dataset categorized 98.8% of cases, with 98.4% specificity and 100% sensitivity, giving an AUC of 0.999 [18].

The TBI was settled with a random forest with a leave-one-out cross-validation system AI-based algorithm that combines data from the corneal deformation response and the corneal tomography to augment the capability to divide normal and modified eyes. The cutoff of 0.79 delivered 100% sensitivity and specificity to identify clinical ectasia designed by KC and very asymmetric ectasia (VAE-E) cases. For the normal topographic eyes from VAE patients, the criteria higher than 0.29 provided 90.4% sensitivity and 96% specificity with an AUC of 0.985. The TBI has a statistically higher AUC than all other tested parameters, including the CBI [17]. Subsequently, different studies established that the TBI was the most sensitive index to confirm mild ectasia [87, 91–93]. Subsequent external validation studies validate that the TBI might identify mild forms of ectasia in very asymmetric ectasia with typical topography (VAE-NT) cases [94]. Recently, a new optimized version of the TBI (TBIv2) has progressed with significantly higher accuracy (0.945) for identifying VAE-NT (84.4% sensitivity and 90.1% specificity; cutoff 0.43) and analogous AUC for clinical ectasia (0.999; 98.7% sensitivity; 99.2% specificity; cutoff 0.8). Furthermore, pondering all cases, the TBIv2 had a higher AUC (0.985) than TBIv1 (0.974) and PRFI (0.972) (Fig. 13) [44, 95].

A novel biomechanical KC staging parameter 'E' [96], based on the Corvis biomechanical factor (CBiF), was developed by Seitz and coworkers [97]. The ‘E’ supplied a measure for diverse stages of the biomechanical destabilization of the cornea, being additive to the tomographic Belin’s ABCD ectasia/KC staging (Fig. 6) [98, 99]. Integrating biomechanical E-staging aims to attain biomechanical staging, rather than detecting KC. The evaluation of tomographic (A, B, and C), DCVA (D), and the biomechanical parameter (E) may offer clinical benefits over using either alone [96]. Subsequent research is needed to establish the clinical feasibility of applying such information as predictive tests for prognostic purposes.

The application of Brillouin spectroscopy for the diagnosis of ECD has been investigated; this technology allows for the biomechanical characterization of the cornea, crystalline lens, and sclera [100–102]. It's the principle that light scattering is based on the interaction of light and the intrinsic acoustic waves within the tissue [103]. Ex vivo ectatic corneas have a significantly smaller Brillouin frequency shift than normal corneas [104]. This technology may identify a focal weakening in the elastic modulus and find significant differences between Brillouin measurements in the cone region and other corneal loci in vivo*,* allowing earlier disease detection [103].

Dupps and collaborators demonstrated the ability of phase-decorrelation optical coherence tomography (PhD-OCT) to detect stromal crosslinking changes in porcine and human corneas. PhD-OCT applies the theory of dynamic light scattering to spatially resolve endogenous random motion by calculating the decorrelation rate, Gamma, from the OCT signal with less dependence on intraocular pressure (IOP) [105]. Hafezi and collaborators developed quasi-static optical coherence elastography (OCE) to investigate corneal biomechanical behavior and monitor the changes after crosslinking procedures [106]. Also, polarization-sensitive optical coherence tomography (PS-OCT), developed by Sinha-Roy and collaborators, has a promising ability to evaluate the arrangement of collagen fibrils with ultrahigh-resolution [107, 108]. Additional clinical testing is required for these encouraging diagnostic tools.

### Ocular wavefront analysis

Ocular aberrometry is a diagnostic tool that offers valuable information about the eye's refractive status [109]. Although commonly used to look into low and higher-order aberrations and in planning wavefront-guided refractive surgery, studying higher-order aberrations has raised considerable interest in corneal diseases, including KC [110].

Irregular astigmatism resulting from corneal distortion is associated with a decrease in the optical quality of the cornea and a substantial increase in higher-order aberrations [111]. The ocular wavefront helps understand patients' complaints better. Eventually, it improves the quality of vision with the use of glasses or contact lenses, which is the current objective of refractive, elective, and therapeutic surgery to provide quality vision, and thus improve the patient’s quality of life. Furthermore, investigators have proposed using this technology to enhance the detection of milder forms of the disease [112].

### Genetics and molecular biology

The genetic description of ECDs is a contest. KC improvement has been related to several genes, including VSX-1, miR-184, DOCK9, SOD1, RAB3GAP1, and HGF [113]. The documentation of at least 17 genomic loci in KC patients revealed the genetic heterogeneity of the disease [19], complemented by the description of both autosomal dominant and recessive patterns [114].

In tandem, molecular biology can play a significant part in the diagnosis and classification of KC, which may eventually change the definition of the disease. Histopathologic studies described molecular and cellular changes related to the pathogenesis of KC, including extracellular matrix degeneration [115]. Some nucleotide polymorphisms of the Lysyl oxidase, an essential component of the extracellular matrix via enzymatic reaction, could potentially be used for KC risk prediction [29].

## Conclusion

Multimodal imaging is essential for a comprehensive evaluation of ECDs, including diagnosis, classification, staging, prognosis, individualized treatment planning, and clinical follow-up. Such knowledge is fundamental given the profound transformation in managing such diseases. Assessing ectasia risk before refractive surgery aims not only to identify candidates with mild keratoconus but preventing iatrogenic ectasia involves the characterization of the inherent susceptibility to biomechanical failure, which represents a second paradigm shift related to the diagnostic ability for ECD. The understanding of corneal structure must be considered along with the relational impact of the procedure for quantifying the risk of ectasia [58]. While the evolution in corneal imaging diagnosis over the last 30 years has been enormous, this evolution continues. In the future, AI will empower enhanced data integration, including corneal tomography and biomechanical assessments, along with epithelium segmental layered epithelium, microlayer (Bowman) tomography, axial length, ocular wavefront, and other tests such as molecular biology, and genetics. This approach will increase efficiency and safety [86], facilitating clinical decision-making. The concept is to expand the application from the diagnosis to clinical and surgical management, propelling diagnostics into precision-driven ophthalmic care for patients with ECDs.

## Data Availability

Not applicable.
